# Tyrosine kinase 2 modulates splenic B cells through type I IFN and TLR7 signaling

**DOI:** 10.1007/s00018-024-05234-y

**Published:** 2024-04-29

**Authors:** Irene Bodega-Mayor, Pablo Delgado-Wicke, Alejandro Arrabal, Estíbaliz Alegría-Carrasco, Ana Nicolao-Gómez, Marta Jaén-Castaño, Cristina Espadas, Ana Dopazo, Enrique Martín-Gayo, María Luisa Gaspar, Belén de Andrés, Elena Fernández-Ruiz

**Affiliations:** 1 Molecular Biology Unit, Hospital Universitario de La Princesa and Research Institute (IIS-Princesa), Madrid, Spain; 2 Present Address: Immunobiology Unit, Centro Nacional de Microbiología, Instituto de Salud Carlos III, Majadahonda, Madrid, Spain; 3 Genomics Unit, Centro Nacional de Investigaciones Cardiovasculares, Madrid, Spain; 4 CIBER de Enfermedades Cardiovasculares (CIBERCV), Madrid, Spain; 5 Immunology Department, Hospital Universitario de La Princesa and IIS-Princesa, Madrid, Spain; 6 Faculty of Medicine, Universidad Autónoma de Madrid, Madrid, Spain

**Keywords:** Tyrosine kinase 2 (TYK2), TYK2-deficient mice, Aged B cells, Marginal zone B cells, Follicular B cells, Toll-like receptor 7 (TLR7), Type I IFN, IFNα, β receptor

## Abstract

Tyrosine kinase 2 (TYK2) is involved in type I interferon (IFN-I) signaling through IFN receptor 1 (IFNAR1). This signaling pathway is crucial in the early antiviral response and remains incompletely understood on B cells. Therefore, to understand the role of TYK2 in B cells, we studied these cells under homeostatic conditions and following in vitro activation using *Tyk2*-deficient (*Tyk2*^*−/−*^) mice. Splenic B cell subpopulations were altered in *Tyk2*^−/−^ compared to wild type (WT) mice. Marginal zone (MZ) cells were decreased and aged B cells (ABC) were increased, whereas follicular (FO) cells remained unchanged. Likewise, there was an imbalance in transitional B cells in juvenile *Tyk2*^−/−^ mice. RNA sequencing analysis of adult MZ and FO cells isolated from *Tyk2*^−/−^ and WT mice in homeostasis revealed altered expression of IFN-I and Toll-like receptor 7 (TLR7) signaling pathway genes. Flow cytometry assays corroborated a lower expression of TLR7 in MZ B cells from *Tyk2*^*−/−*^ mice. Splenic B cell cultures showed reduced proliferation and differentiation responses after activation with TLR7 ligands in *Tyk2*^*−/−*^ compared to WT mice, with a similar response to lipopolysaccharide (LPS) or anti-CD40 + IL-4. IgM, IgG, IL-10 and IL-6 secretion was also decreased in *Tyk2*^*−/−*^ B cell cultures. This reduced response of the TLR7 pathway in *Tyk2*^−/−^ mice was partially restored by IFNα addition. In conclusion, there is a crosstalk between TYK2 and TLR7 mediated by an IFN-I feedback loop, which contributes to the establishment of MZ B cells and to B cell proliferation and differentiation.

**Supplementary Information:**

The online version contains supplementary material available at 10.1007/s00018-024-05234-y.

## Introduction

B lymphocytes are an essential component of the humoral immune response. They have a diverse origin: adult B-1 cells arise almost entirely from fetal precursors, while B-2 cells derive from B cell progenitors in the bone marrow [1]. B-2 cells migrate from the bone marrow as immature cells, and they colonize the spleen and lymph nodes as newly formed or transitional B cells. Afterward, they differentiate into follicular (FO) and marginal zone (MZ) B cells [2] and, in aged and autoimmune prone mice, into the so-called aged B cells (ABC) [3]. FO B cells enter the germinal center to participate in T cell-dependent immune reactions, undergoing class switching and somatic hypermutation to produce high-affinity antibodies and memory B cells [4]. MZ B cells are the first line of defense against blood-borne pathogens and rapidly produce low-affinity IgM antibodies against encapsulated bacterial and polysaccharide antigens, independently of T cells [5, 6]. In addition to B cell receptor (BCR), B cell subpopulations express other receptors such as Toll-like Receptors (TLRs) that modulate their ability to respond to pathogen antigenic patterns [7–9].

Janus kinases (JAKs) are a family of non-receptor tyrosine-kinase proteins with four members (TYK2, JAK1-3) that are associated with cytokine and growth factor receptors [10]. TYK2 was initially identified as essential for IFNα signaling through the IFNα/β receptor alpha chain (IFNAR1), while JAK1 was associated with the IFNAR beta chain (IFNAR2) [11, 12]. IFNAR engagement results in JAK1 and TYK2 activation leading to the formation of the transcription factor complex IFN-stimulated gene factor 3 (ISGF3), which is composed of signal transducers and activators of transcription (STAT1 and STAT2) and IFN-regulatory factor 9 (IRF9) [13]. This complex translocates to the nucleus to promote transcription of IFN-stimulated genes [12, 14], whose products establish an antiviral state in the cell [15, 16]. Type I IFNs exert antiproliferative and immunomodulatory effects essential to control viral infection. In addition, TYK2 signals through several cytokine receptors as IL-12Rβ1, IL-10Rβ, IL-6Rα, IL-11Rα, CNTFRα and IL-13Rα1 [17]. The impairment of JAK/STAT signaling has been related with susceptibility to infection, autoimmune diseases and deregulated leukocyte proliferation and survival [18].

Patients with TYK2 deficiency have defects in the signaling of the above mentioned cytokines leading to a high susceptibility to intracellular viral and bacterial infections [19–23]. In contrast, TYK2 overexpression has been described in lung leukocytes obtained from SARS-CoV-2 infected patients, where is directly related to high rates of vital risk [24]. Protective TYK2 genetic variants have been identified in autoimmune diseases [25, 26], but their relevance in other pathologies such as hematopoietic malignancies is still uncertain [27]. Consistent with the human data, TYK2-deficient (*Tyk2*^−/−^) mice exhibit defective IL-12, IL-23 and type I IFN signaling leading to a down-regulation of Th1 and Th17 cell subpopulations and to susceptibility to viruses and encapsulated bacteria [28–31]. In addition, *Tyk2*^−/−^ mice exhibit reduced inflammatory responses in several autoimmune disease models [26, 32–34] and are resistant to LPS-induced septic shock [35]. This resistance was thought to be mediated primarily by a suppression of the IFNβ-dependent pathway. Yet, TYK2 has been described as an upstream regulator of CASP11-induced pyroptosis, which is required for LPS-induced lethality in mice [36]. Furthermore, an inhibition of IL-27 and an increase in IL-10 production in *Tyk2*^−/−^ peritoneal macrophages have been linked to the anti-inflammatory and immunosuppressive phenotype induced by TYK2 deficiency [37, 38].

The way in which TYK2 deficiency affects T cell-dependent B cell differentiation from naive B cells to plasma and memory cells has been studied with T helper follicular cells and splenic B cells derived from patients with monogenic TYK2 mutations [39]. The effect of TYK2 on IL-7-dependent B cell lymphopoiesis, apoptotic responses to IFN-I and mitochondrial respiration maintenance in bone marrow-derived pro-B lymphocytes has been described in mice [40–42], whereas the role of this tyrosine kinase in T cell independent B cell responses remains incompletely understood. Therefore, herein we characterized the B cell subpopulations in immune organs of *Tyk2*^−/−^ mice. We found that under homeostatic conditions the total number of B cells was not altered. However, the splenic B cell distribution showed an increase of ABC and a decrease of MZ cells, while FO cells remained unchanged. Differentially expressed gene (DEG) analysis of isolated MZ and FO cells from *Tyk2*^−/−^ compared to WT mice in homeostasis shared an inhibited type I IFN-dependent pathway, which linked TYK2 deficiency with TLR7 down-regulation. Accordingly, in vitro stimulation of *Tyk2*^−/−^ splenic B cells with TLR7 ligands (TLR7L) demonstrated a defect in proliferation and differentiation, while their response to TLR4 ligands or anti-CD40 + IL-4 was comparable to that of WT B cells. Furthermore, in vitro TLR7L-stimulated *Tyk2*^−/−^ splenic B cells showed a diminished humoral response. Taking these results together, we propose that impaired signal transduction through TLR7 in *Tyk2*^−/−^ mice affects splenic B cells, in particular the establishment and differentiation of MZ cells, and contributes to immunosuppression.

## Material and methods

### Mice

Three to four-month-old male and female C57BL/6N (WT) and B6N.129P2-Tyk2KO tm1Biat (*Tyk2*^−/−^) mice (on a C57BL/6N background) [28] (kindly provided by Dr. M. Müller, Institute of Animal Breeding and Genetics, University of Veterinary Medicine, Vienna, Austria) were bred and maintained in the animal facilities at the School of Medicine of the Universidad Autónoma de Madrid. All experiments were performed in accordance with the National RD 53/2013 and European Union 2010/63/EU directive and under the EU and National Animal Care guidelines. All protocols were approved by Consejería de Medio Ambiente de la Comunidad de Madrid (PROEX 353.8/21).

### Flow cytometry and sorting

Cell pellets were obtained from spleen, peripheral blood mononuclear cells (PBMCs), axillary and inguinal lymph nodes, bone marrow (femur and tibia), thymus, Peyer’s patches or peritoneal washed cells. Cell homogenates from the spleen were treated with ACK lysing buffer (Gibco™, Thermo Fisher Scientific, Waltham, MA, USA) to lyse the erythrocytes. In the case of PBMCs, a density gradient (Lymphoprep™, Cedarlane Laboratories, Burlington, ON, Canada) was performed. The cells were then suspended in staining buffer (2.5% Fetal Calf’s Serum) in Dulbecco’s Phosphate buffered saline (DPBS, BioWhittaker™, Lonza Group, Basel, Switzerland). Non-specific antibody binding was blocked by incubation with anti-Fc-Block (clone 2.4G2: BD Biosciences, San Jose, CA, USA). The single cell suspensions were stained with the specific mAbs listed in Table S1 and analyzed by flow cytometry. For intracellular staining, after Fc-blocking and surface and live/dead staining (LIVE/DEAD™ kit, Invitrogen™, Thermo Fisher Scientific), the cells were fixed using the Cytofix/Cytoperm kit (BD Biosciences) prior intracellular staining. Isotype and fluorescence minus one (FMO) controls were included. Doublets were discriminated using the FSC-H versus FSC-W strategy, and dead cells were discharged by staining with propidium iodide (PI) in fresh cell preparations, unless otherwise indicated, before acquisition on an LRS Fortessa X-20 cytometer (BD Biosciences). When indicated, cells were purified (over 95% purity) using a FACSAria-I (BD Biosciences) cell sorter. Cytometry data were analyzed using the FlowJo v10.8.1 (TreeStar, Ashland, OR, USA) and DIVA v8.0 (BD Biosciences) software packages. At least 1 × 10^5^ live cells were analyzed in each sample and the cell populations were identified as indicated in Table S2. PE Annexin-V staining was performed following manufacturer’s instructions (Immunostep, Salamanca, Spain).

### Gene expression analysis by RNA-Seq and computational data analysis

Total RNA was isolated from sorted FO, MZ and ABC using RNeasy^Ⓡ^ kits (Qiagen, Hilden, Germany). Quantity and integrity of each RNA sample was checked using a Bioanalyzer at the CNIC Genomics Unit (Madrid, Spain). A total of 0.5 ng of RNA from MZ cells was used to generate barcoded RNA-seq libraries using the NEBNext Single Cell/Low Input RNA Library Prep Kit for Illumina (New England Biolabs, Ipswich, MA, USA) according to manufacturer’s instructions. First, cDNA strand synthesis was performed and then amplified by PCR followed by fragmentation. Next, cDNA ends were repaired and adenylated. The NEBNext adaptor was then ligated followed by second strand removal, uracil excision from the adaptor and PCR amplification. In the case of FO cells, 200 ng of total RNA were used to generate barcoded RNA-seq libraries using the NEBNext Ultra II Directional RNA Library Prep Kit (New England Biolabs). Poly A + RNA was purified using poly-T oligo-attached magnetic beads followed by fragmentation and then, first and second cDNA strand synthesis. Second strand was synthesized with U ribonucleotide instead of T. Next, cDNA 3' ends were adenylated and the adapters were ligated. Second strands were fragmented by USER enzyme that cuts the uracils to preserve the directionality of the original RNA. Once the libraries were generated, they were amplified by PCR. The size of the libraries was checked using the Agilent 2100 Bioanalyzer High Sensitivity DNA chip and their concentration was determined using the Qubit^Ⓡ^ fluorometer (Thermo Fisher Scientific). Libraries were sequenced on a HiSeq 4000 and processed with RTA v1.18.66.3. FastQ files for each sample were obtained using bcl2fastq v2.20.0.422 software (Illumina, San Diego, CA, USA). Sequencing reads were aligned to the mouse reference transcriptome (GRCm38 V90) and quantified with RSem v1.3.1 [43]. Raw counts were normalized with TPM (Transcripts per Million) and TMM (Trimmed Mean of M-values) methods, transformed into log_2_ expression (log_2_[rawCount + 1]) and compared to calculate fold-change and corrected *P*-value. Only those genes expressed with at least 1 count in a number of samples equal to the number of replicate samples of the condition with fewer replicates were considered. For functional analysis, those genes with |log_2_FC|> 0.58 and unadjusted *P*-value < 0.05 were considered, although we also calculated the Benjamini and Hochberg correction for all genes. Finally, heatmaps were generated with Morpheus online tool from Broad Institute (https://software.broadinstitute.org/morpheus), while pathway analysis and visualization of gene networks for each DEG list were performed using Ingenuity Pathway Analysis (Qiagen) and NetworkAnalyst [44] software, respectively.

### RT-qPCR assays

Total RNA was extracted from purified FO, MZ, ABC B cells or from splenic B cell cultures using TRIsure™ (Bioline, London, UK) following the manufacturer’s instructions. Reverse-transcriptase polymerase chain reaction (RT-PCR) was performed using Superscript^Ⓡ^ Vilo™ cDNA kit (Applied Biosystems™, Thermo Fisher Scientific). cDNA expression was evaluated by quantitative PCR (qPCR) performed in triplicates using TaqMan Fast-Advanced Master Mix (Applied Biosystems™, Thermo Fisher Scientific) in a CFX384 Real-Time System (BioRad, Hercules, CA, USA). The TaqMan assays used were: Mm99999915_g1 (*Gapdh*), Mm00446590_m1 (*Tlr7*), Mm00439531_m1 (*Stat1*), Mm00438023_m1 (*Casp1*), Mm01176201_mH (*Zbtb44*), Mm00510242_m1 (*Mzb1*), Mm01175819_m1 (*Ets1*), Mm00600614_m1 (*Jak1*), Mm00439544_m1 (*Ifnar1*), Mm00476128_m1 (*Prdm1*/*Blimp1*), Mm00457357_m1 (*Xbp1*) and Mm00507774_m1 (*Aicd*) (Thermo Fisher Scientific). For each sample, relative mRNA levels were determined using the comparative Ct (2^-ΔΔCt^) method (samples normalized to their *Gapdh* content).

### Proliferation and differentiation analysis

Splenic cells were obtained after mechanical disaggregation of the spleen over a 40 μm cell strainer (Falcon^Ⓡ^, Corning, NY, USA). Erythrocytes were removed by incubation with 1 mL of ACK lysis buffer for 2 min and washing twice in DPBS 1 X (Lonza Group). For B cell enrichment, cells were incubated with anti-Thy1.2 (clone J1J, in house reagent) for 20 min at room temperature (RT), washed to discard antibody excess and subsequently incubated with rabbit complement for 30 min at 37 °C (Cedarlane Laboratories), as described [45]. After T cell depletion, non-adherent cells were collected and washed. The recovered cells were enriched in B lymphocytes (> 95% CD19^+^). They were labelled with cell trace reagent (CellTracker™ BMQC Violet, Thermo Fisher Scientific), following the manufacturer’s instructions, and were seeded into flat-bottom 96-well culture plates (1 × 10^6^ cells/mL). They were cultured for 6–96 h at 37 °C and 5% CO_2_ in complete RPMI 1640 (10% heat-inactivated FCS, 2 mM l-glutamine, 1 mM pyruvate, 50 mM 2-ME, 10 mM HEPES and antibiotics) in the presence of different stimuli: CL097 (2 μg/ml; InvivoGen, San Diego, CA, USA), Imiquimod/R837 (2 μg/ml; InvivoGen), IFNα (20 ng/ml; Biolegend, San Diego, CA, USA), LPS (25 μg/ml; Sigma-Aldrich, St. Louis, MO, USA) and anti-CD40 (10 μg/ml; HM40-3; eBioscience™, Thermo Fisher Scientific) with IL-4 (150 ng/ml; PeproTech, Thermo Fisher Scientific). Where indicated, the splenic B cell cultures were incubated with IFNα (20 ng/ml). Then, cells were washed with fresh cell medium and cultured for 6 h with the different stimuli. Proliferation was assessed by quantifying the cell trace dye-dilution representing cell divisions. B cell differentiation to plasma cells was studied by labeling with anti-CD138.

### Cytokine analysis

Cytometric 13-multiplex cytokine bead arrays (CBA) were used to quantify IL-23, IL-1α, IFN-γ, TNF-α, CCL2 (MCP-1), IL-12p70, IL-1β, IL-10, IL-6, IL-27, IL-17A, IFN-β, GM-CSF (Biolegend) in culture samples. The determinations were performed in duplicate according to the manufacturer’s instructions. Samples were run on a CANTO I or FORTESSA (BD Biosciences) cytometer. The calibration curves were above r^2^ 0.97.

### Immunoglobulin (Ig) determination by ELISA

IgM, IgG and IgA were measured by ELISA in the supernatants obtained from B cell splenic cultures. Briefly, flat-bottom 96-well plates (Nunc, Rochester, NJ, USA) were coated with unlabeled goat-anti mouse total Ig (10 µg/ml; SouthernBiotech, Birmingham, AL, USA) and they were then blocked with 0.5% gelatin in phosphate buffered saline (PBS, BioWhittaker™, Lonza Group). Frozen supernatants were thawed and serial dilutions were prepared in the ELISA buffer (see below) and were plated in duplicate (at 50 μL/well). After overnight (4ºC) incubation, the plates were washed in PBS, 50 μL/well of biotinylated goat anti-mouse-IgM, -IgG or -IgA (SouthernBiotech) were added and the plates were incubated for 1 h. Subsequently, 50 μL/well of streptavidin-peroxidase (SouthernBiotech) were added and incubated for 30 min at RT. All the regents were previously tested and were diluted in 0.1% Tween-20 0.5% gelatin PBS. The ELISA plates were then revealed with 0.5 M O-phenylenediamine (Sigma-Aldrich) and the reaction was stopped with 3 N H_2_SO_4_. OD values were obtained at 450 nm in a Multiskan FC ELISA-reader (Thermo Fisher Scientific). Standard curves were generated using purified mouse IgM, IgG and IgA (SouthernBiotech), and the Ig concentrations were calculated using the GraphPad Prism 8.0 software.

### Statistical analysis

All statistical analyses were performed using GraphPad Prism 8.0 software after testing the normality of the data distributions with the Kolmogorov–Smirnov and D’Agostino-Pearson tests. The data are presented as the means ± SEM. Comparisons were performed using two-tailed unpaired Student's t-tests and ANOVA for multiple comparisons. *P < 0.05, **P < 0.01, ***P < 0.001.

## Results

### *Tyk2* deficiency alters the number of MZ B cells and ABC

**Fig. 1:**
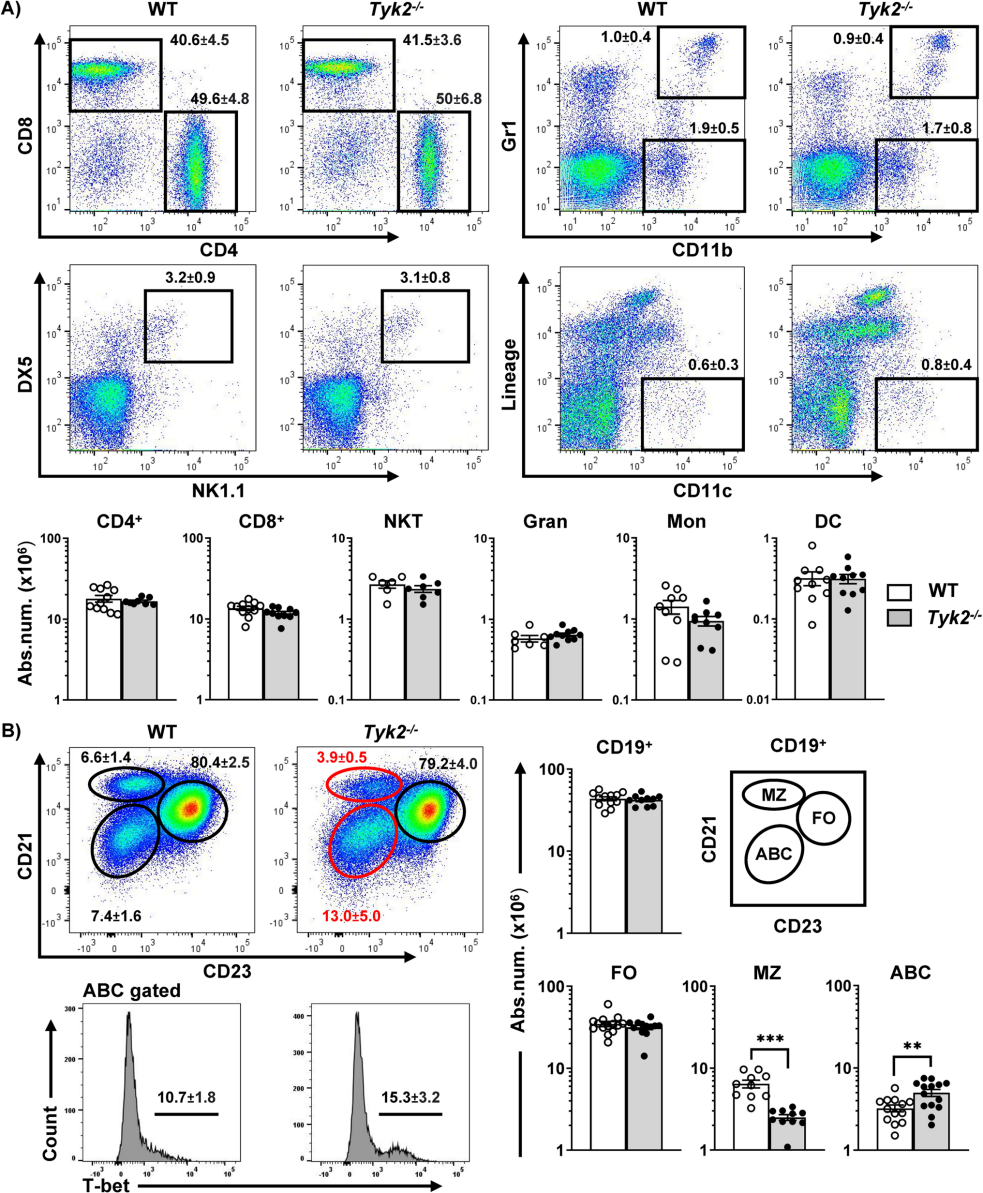
Altered distribution of splenic MZ cells in *Tyk2*^−/−^ mice under homeostatic conditions. Splenic cell suspensions from WT (white) and *Tyk2*^−/−^ mice (grey) were prepared, counted and stained for flow cytometry analyses (see Materials and Methods for details). **A** Representative dot plots of 3-month-old WT and *Tyk2*^−/−^ mice are shown on the left, with numbers inside the plots displaying the frequencies of each population indicated in the boxes (mean ± SEM; n = 6–11). Absolute numbers of live spleen NKT cells (CD3^+^DX5^+^NK-1.1^+^), granulocytes (Gran, CD11b^+^Gr-1^+^), monocytes (Mon, CD11b^+^Gr-1^−^), dendritic cells (Lineage: Gr-1^−^CD19^−^DX5^−^CD90.2^−^, CD11c^+^) and T cells (CD3^+^CD4^+^ and CD3^+^CD8^+^) determined by flow cytometry are shown. **B** Splenic B cells were electronically gated using CD19, and MZ, FO and ABC cell compartments were identified as CD21^++^CD23^lo^ (MZ, ellipse), CD21^+^CD23^++^ (FO, circle) and CD21^lo^CD23^lo^T-bet^+^ (ABC, ellipse). Representative dot plots of 3-month-old WT and *Tyk2*^−/−^ mice are shown. The numbers inside the plots are the frequencies of each population (mean ± SEM; n = 10–14) among CD19^+^ cells. The graphs show the absolute number of spleen of B cells (CD19^+^), MZ, FO and ABC cells. Scales are logarithmic. Data in the graphs in A and B are shown as scatter dot plots representing individual values, and the bars are mean ± SEM depicted for each group (n = 10–14 for B cells, n = 8–11 for CD4 and CD8 cells, n = 6–7 for NKT cells and n = 7-10 for myeloid cells). These numbers were calculated from the frequencies obtained from flow cytometry of each population. Group comparisons were made using a two-tailed Student t-test: **P < 0.01, ***P < 0.001

**Fig. 2:**
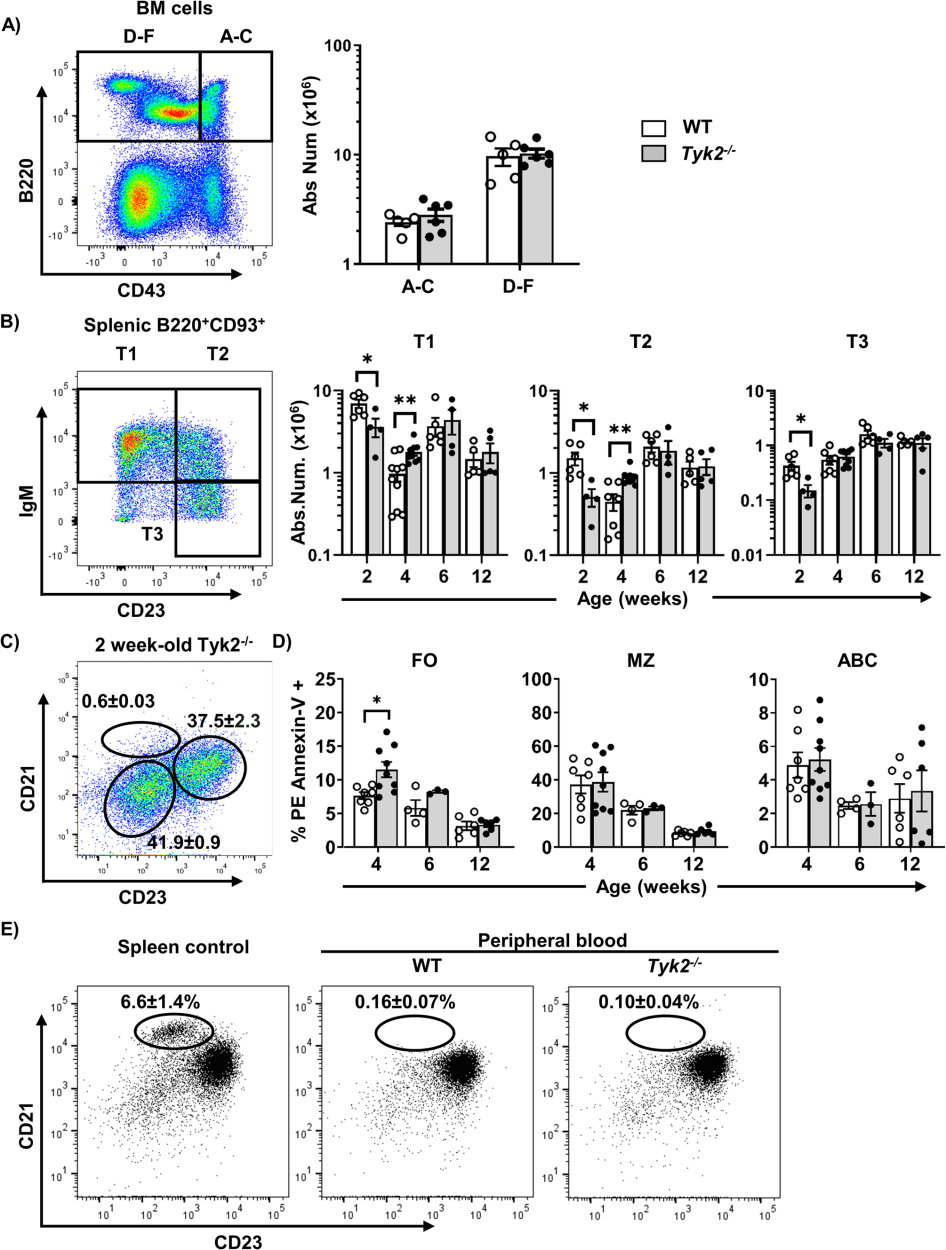
Immature B cell progenitors and transitional B cells in *Tyk2*^−/−^ mice under homeostatic conditions. **A** Bone marrow cells from adult WT and *Tyk2*^−/−^ mice were processed for flow cytometry studies using anti-CD43 and anti-B220 to identify B cell progenitors [46] (see Table S2). Left, representative dot plot of the staining obtained in WT mice; Right, quantitation of CD43^+^ (fractions A-C) and CD43^−^ (fractions D-F) cells in WT and *Tyk2*^−/−^ mice (n = 5–6). **B** Splenic cells were stained with anti-B220, anti-CD93, anti-IgM and anti-CD23 in order to trace transitional B cells (T1-T3) as described [47]. Left, representative dot plot of IgM and CD23 staining on electronically gated B220^+^CD93^+^ cells, in splenic WT mice. Right, quantification of absolute number of spleen transitional (T1-T3) B cells on infant and young mice (n = 4–10). **C** Absence of MZ cells in 2 week-old *Tyk2*^*−/−*^ mice (n = 4). **D** Apoptotic cells were determined by flow cytometry using PE Annexin-V on splenic B cell suspensions stained as in Fig. 1B to identify MZ, FO and ABC cells. Data are presented as the percentage of PE Annexin-V + cells in each population (n = 3–9). **E** Identification of MZ cells in peripheral blood suspensions after staining with anti-CD19, anti-CD21 and anti-CD23 as in Fig. 1B. Representative dot-plots of adult WT spleen, and adult WT and *Tyk2*^−/−^ peripheral blood cells (PBMC) are shown; numbers inside the plots are the mean ± SEM (n = 3–8). Data in the bar graphs in A, B and C are presented as individual values in the scatter plots, with the bars showing the mean ± SEM. These numbers were calculated from the frequencies of each population. Group comparisons were performed with a two-tailed Student t-test: *P < 0.05, **P < 0.01

The splenic cellular content of *Tyk2*^−/−^ mice exhibited similar relative and absolute numbers of B cells, NK cells, myeloid cells and T cells compared to control mice (Fig. 1A), although the distribution of B cell subsets was altered (Fig. 1B). Splenic B cell subpopulations [2] can be distinguished by the surface markers CD21 and CD23, into FO (CD21^+^CD23^+^), MZ (CD21^++^CD23^lo/−^) and ABC (CD21^−^CD23^−^), being ABC cells further identified on the basis of T-bet expression (Fig. 1B). In the absence of TYK2, FO B cells remained unchanged, MZ B cells were significantly decreased and ABC (T-bet^+^) were overrepresented (Fig. 1B). Cell quantification in peritoneal lavage, lymph nodes, Peyer’s patches and thymus showed no differences in B2, B1 or T cells (Fig. S1). To elucidate whether these differences were linked to defects in bone marrow precursors, we analyzed the B cell progenitor fractions as described [46] (Fig. 2A). No differences were found in any of the bone marrow B cell fractions in *Tyk2*^−/−^ mice compared to controls. We then characterized transitional B cells in the spleen (T1, T2 and T3) on the basis of IgM and CD23 membrane expression of B220^+^CD93^+^ cells [47]. At 2 weeks of age, MZ B cells were not yet formed (Fig. 2C), and T1, T2 and T3 B cells were diminished in *Tyk2*^−/−^ mice. This reduction was overcome at 4 weeks of age, when increased total numbers of T1 and T2 cells were observed (Fig. 2B), while similar numbers were maintained in older mice.

Since MZ B cells were not yet detected at 2 weeks of age (Fig. 2C) as described [48, 49], we performed PE Annexin-V studies on 4-week old juvenile mice [48, 50] to analyze whether apoptosis could explain the reduced numbers of MZ B cells. The results revealed that only FO, but not MZ B cells, had increased levels of apoptosis in *Tyk2*^−/−^ mice (Fig. 2D). Furthermore, to rule out the possibility that MZ B cells were not being retained in the splenic niche, we analyzed their presence in peripheral blood. We observed that there were no MZ B cells in peripheral blood from either *Tyk2*^−/−^ or WT mice discarding differences in migration of these cells (Fig. 2E).

All these results demonstrate that *Tyk2* deficiency alters transitional B cell differentiation in juvenile mice and diminishes splenic MZ B cells without increased apoptosis or aberrant distribution of this population.

### Differential gene expression analysis of isolated *Tyk2*^*−/−*^ MZ and FO B cells shows a common inhibited type I IFN-dependent pathway

To search for candidate genes responsible for the impaired MZ B cell development in *Tyk2*^*−/−*^ mice, we next compared the gene expression pattern of isolated MZ and FO B cells under homeostatic conditions by RNA-seq.

**Fig. 3:**
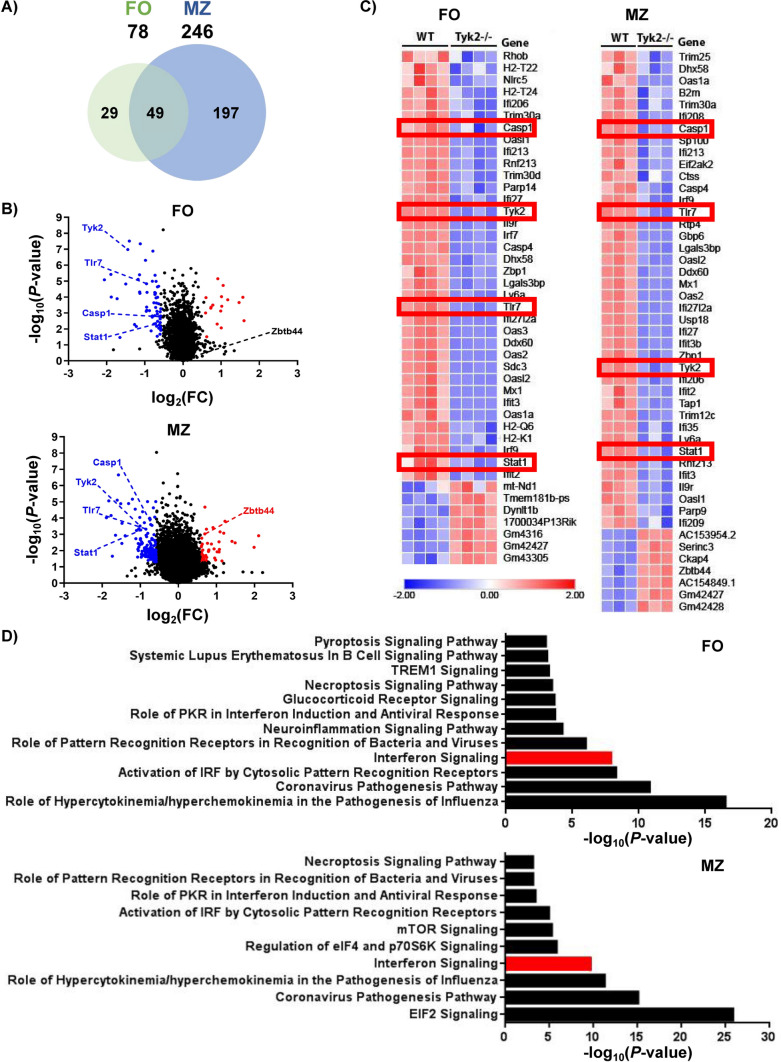
RNA-Seq analysis of gene expression in FO and MZ B cells in homeostasis. FACS-purified FO and MZ cells from WT and *Tyk2*^−/−^ mice were used to prepare total RNA (MZ cells) or mRNA (FO cells) as indicated in the Material and Methods section. **A** Venn diagram showing the number of differentially expressed genes (DEGs) between WT and *Tyk2*^−/−^ mice in each cell subpopulation and those shared by both populations. A gene signature of 49 genes characteristic of *Tyk2* deficiency is observed in both FO and MZ cells. **B** Distribution of DEGs according to FO or MZ cell population plotted as a function of their variation and significance (*P*-value < 0.05). Labeled in red and blue are representative up- and down-regulated genes, respectively. **C** Heatmap of DEGs of the IFN-I signaling pathway in FO and MZ cells. Higher expression levels are indicated in red and lower expression levels in blue. The red boxes highlight genes of interest whose expression was validated. **D** Histograms depicting the main canonical pathways obtained with the Ingenuity Pathway Analysis (IPA) in FO and MZ cells. IFN-I signaling pathway was identified as significantly deregulated (labeled in red). *P*-value < 0.05 for FO and a false discovery rate (FDR) < 0.05 for MZ B cells

A total of 246 genes were differentially expressed compared to WT mice in *Tyk2*^−/−^ MZ B cells *vs* the 78 found in *Tyk2*^−/−^ FO B cells (Fig. 3, Tables S3 and S4). Among the differentially expressed genes (DEGs), 49 were shared by both types of B cells and were mainly related to type I IFN-signaling (Fig. 3C, D and Table S5). In addition, genes involved in necroptosis (*Casp1*, *Irf9, Stat1* and *Zbp1*) and pyroptosis (*Casp1, Casp4* and *Tlr7*) signaling pathways were also affected in MZ and FO B cells. Furthermore, in the case of MZ B cells most of the genes deregulated by *Tyk2* deficiency corresponded to the EIF2 signaling pathway, which is involved in the stress-induced regulation of translation caused by viruses and bacteria [51, 52].

**Fig. 4:**
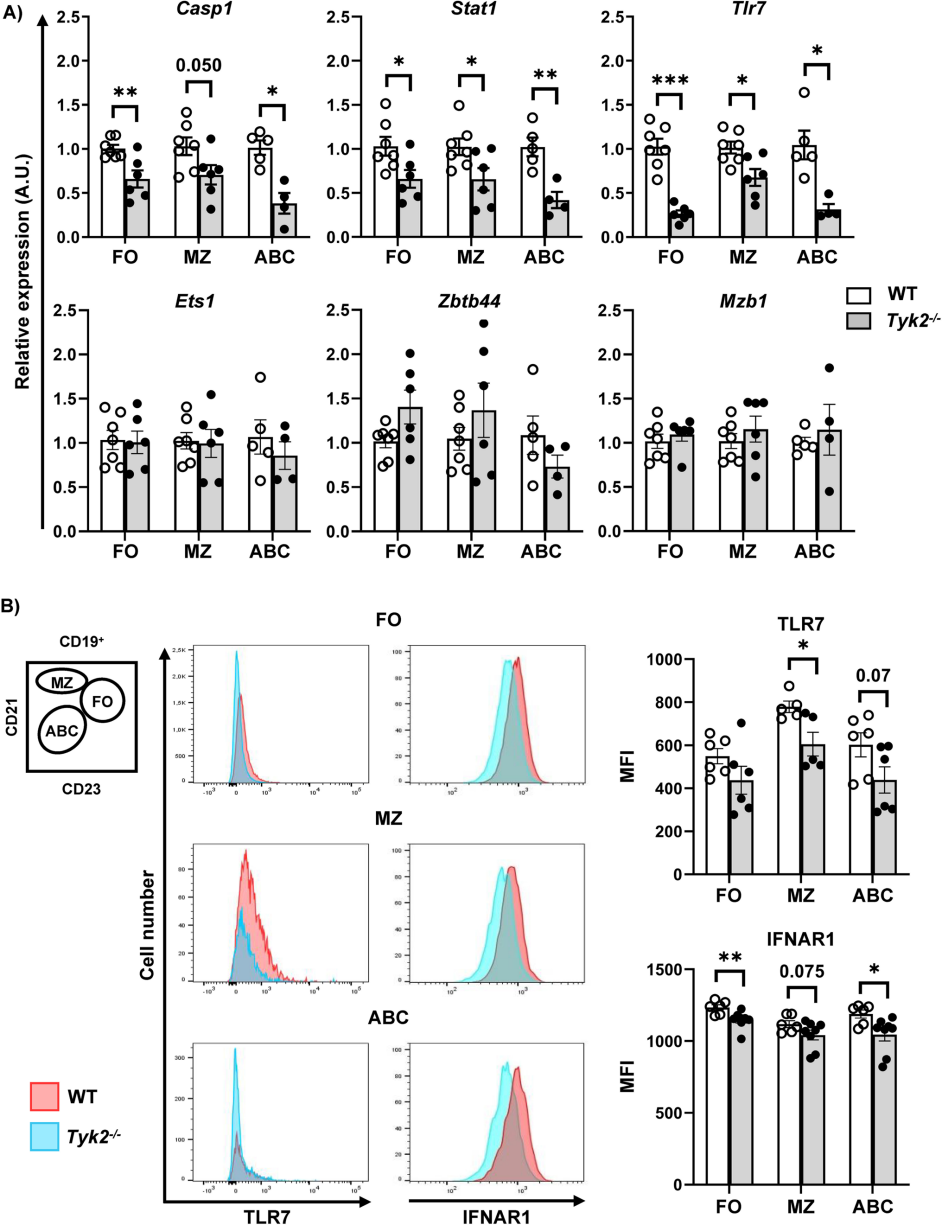
Validation of DEGs data by qPCR and flow cytometry. **A** RNA preparations extracted from FACS-purified splenic cell samples of FO, MZ and ABC cells were analyzed by qPCR as detailed in the Material and Methods section. The bar plots show the relative expression of *Casp1*, *Stat1, Tlr7, Ets1, Zbtb44,* and *Mzb1* in the splenic B cell subpopulations. For each sample, relative mRNA levels were determined by RT-qPCR using the 2^-ΔΔCt^ method normalized to *Gapdh*. Data represent individual values (empty dots, WT samples; black dots, *Tyk2*^−/−^ samples) and mean ± SEM (n = 4–7; white bars WT mice and gray bars *Tyk2*^−/−^ mice). **B** Left, schematic representation of a dot plot of splenic B cells to identify B cell subpopulations, as shown in Fig. 1. Middle, overlaid histograms corresponding to intracellular TLR7 expression (left) and surface IFNAR expression (right) in FO, MZ and ABC subpopulations of WT (red) and *Tyk2*^−/−^ (blue) splenic samples. Right, the bar graphs show the median fluorescence intensity (MFI) of TLR7^+^ or IFNAR^+^ cells, in WT (white) and *Tyk2*^−/−^ (grey) B cells. Data are presented as individual values (dots). Bars show the mean ± SEM (n = 5–8). Group comparisons were performed with a two-tailed Student t-test: *P < 0.05, **P < 0.01, ***P < 0.001

*Tlr7* was also differentially expressed in FO and MZ B cells, linking *Tyk2* deficiency with endosomal TLR7 sensing and response [53]. We validated these data by performing qPCRs (Fig. 4A) and found that *Casp1*, *Stat1* and *Tlr7* were significantly reduced in the three subpopulations of splenic *Tyk2*^−/−^ B cells. By contrast, *Zbtb44,* which is a transcription factor that may be involved in hematopoiesis, oncogenesis and immune responses [54], did not reach significance in any case. In addition, we evaluated *Mzb1* and *Ets1*, which are involved in plasma cell [55] and hematopoietic cell [56] differentiation, respectively. Their levels did not change in *Tyk2*^*−/−*^ compared to WT B cells. Further, we analyzed intracellular TLR7 expression in FO, MZ and ABC by flow cytometry (Fig. 4B). There was a significant decrease of this receptor in MZ B cells of *Tyk2*^−/−^ mice compared to those of WT mice, whereas non-significant differences were obtained for TLR7 expression in plasmacytoid dendritic cells (DC, data not shown). It has been described that TYK2 sustains the membrane expression of its associated receptor IFNAR1, although there is some controversy between studies in humans and mice [20, 57, 58]. We found by flow cytometry that IFNAR1 expression was lower in FO, MZ and ABC cells of *Tyk2*^−/− ^mice compared to those of WT mice, although this difference did not reach statistical significance in the case of MZ B cells. The discrepancy with previous observations in B220^+^ splenic cells where no differences were detected [58] could be due to the B cell type-specific subpopulations analysed here.

We conclude that *Tyk2* deficiency in splenic B cells is related to a defect in type I IFN signaling that affects the expression of TLR7 and IFNAR1.

### Impaired proliferation and differentiation responses of *Tyk2*^*−/−*^ splenic B cells to TLR7 ligands

**Fig. 5:**
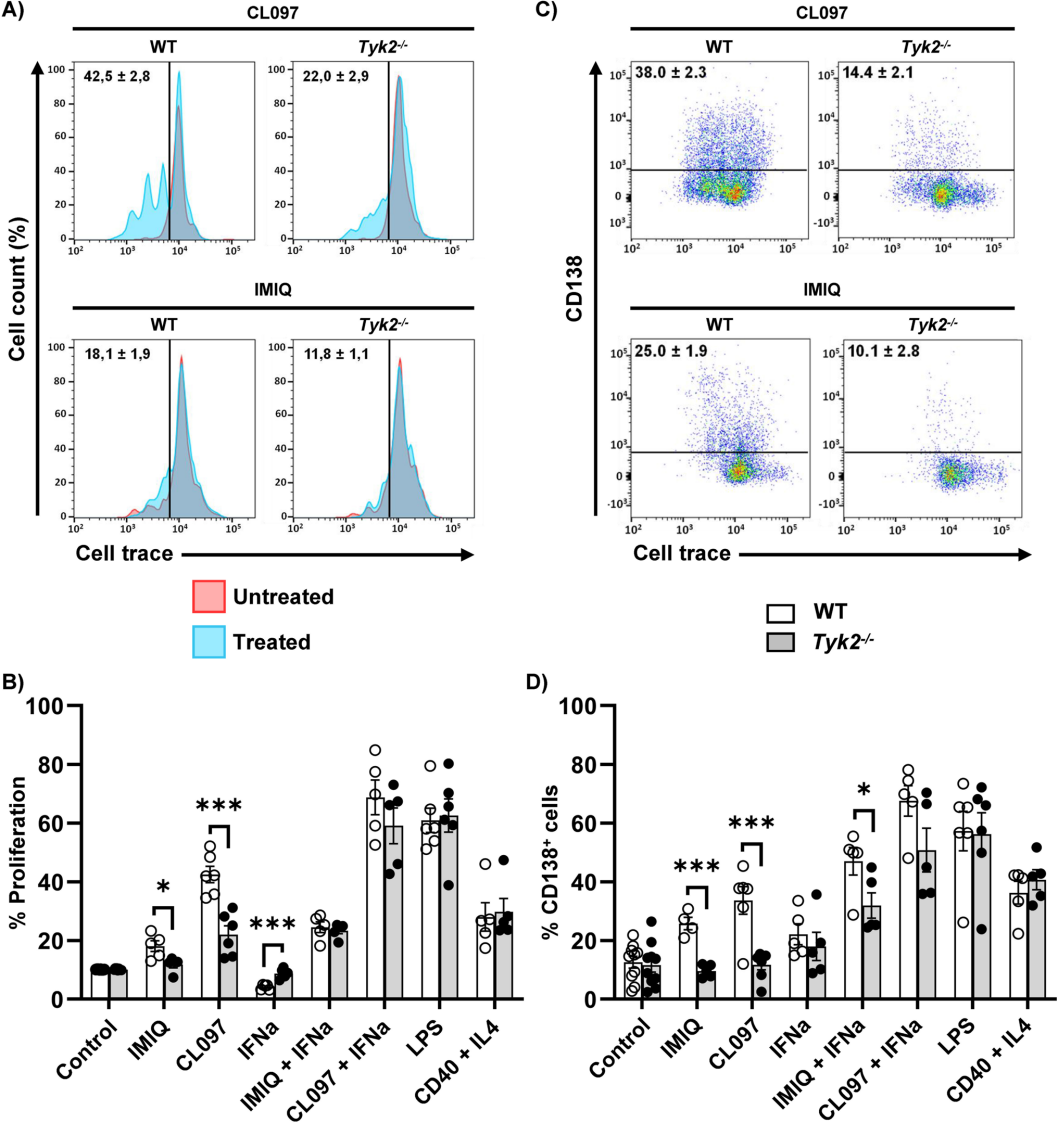
Proliferation and differentiation of splenic B cell cultures. B cells were obtained after depletion of T and plastic-adherent myeloid cells as described in Materials and Methods. The cells were loaded with Cell Trace (CellTracker™) following the manufacturer’s instructions and cultured with CL097 or Imiquimod/R837 (IMIQ, 2 μg/ml) in the absence or presence of IFNα (20 ng/ml), or with LPS (25 μg/ml) or anti-CD40 (10 μg/ml) and IL-4 (150 ng/ml) during 72 h. Cells were then recovered and dye dilution was determined by cytometry after staining with anti-CD138 to assess differentiation. **A** Representative overlaid histograms for CL097 and IMIQ treated cultures (histograms in blue) over untreated cultures (red histogram), displaying the cell trace signal. Proliferation was determined as the percentage of cells with dye diluted by cell division (mean ± SEM; n = 5–6). **B** Quantitation of cell proliferation induced by treatment of cultures with different stimuli for 72 h. **C** Representative dot-plots showing CD138 and cell trace staining on treated cells (untreated cells are not shown). Numbers inside are the percentage of CD138^+^ cells referred to the isotype control (black lines) (mean ± SEM; n = 5–6). **D** Quantitation of CD138^+^ cells after 72 h of culture with different stimuli. Data in B and D are presented as individual values (dots), and the bars show the mean ± SEM (n = 4–10). Statistical analysis was performed by Student's t-test: * *P* < 0.05; ** *P* < 0.01; *** *P* < 0.001

To test whether the low expression of the *Tlr7* gene in *Tyk2*^*−/−*^ mice also entailed a deficient response to its ligands, we performed splenic B cell cultures in which T cells and monocytes had been depleted, in the presence of TLR7L (Imiquimod; IMIQ) and TLR7/TLR8L (CL097) for 72 h (Fig. 5). As positive controls, LPS and anti-CD40 + IL-4 were used to stimulate the innate TLR4-dependent and T cell-dependent responses, respectively. Given that TLR7 expression is induced by type I IFN [59], we added, where indicated, IFNα to splenic B cell cultures (Fig. 5B, D). The effect on proliferation was evaluated by a dye-dilution method after 72 h (Fig. 5A, B). WT B cells stimulated with IMIQ and CL097 showed higher proliferation than *Tyk2*^−/−^ B cells, whereas no differences were observed when B cells were stimulated with LPS or anti-CD40 + IL-4. Plasmablast differentiation was studied by CD138 surface expression (Fig. 5C, D and Fig. S2). WT and *Tyk2*^−/−^ B cells responded similarly to LPS and anti-CD40 + IL-4. In contrast, plasmablast differentiation in response to TLR7L was higher in WT as compared to *Tyk2*^−/−^ B cells (Fig. 5D). *Tyk2* deficiency was not associated with a lower B cell survival rate at 72 and 96 h of culture according to cell viability analysis with propidium iodide (data not shown).

IFNα significantly inhibited the proliferation of splenic B cells from WT compared to *Tyk2*^−/−^ mice. Indeed, these latter were not inhibited compared to the *Tyk2*^−/−^ unstimulated B cells (Figs. 5B and S2). These data are in agreement with those described in bone marrow-derived IL-7-stimulated adult B cells [40] and pro-B cells [41]. When IFNα was added to TLR7L, proliferation was upregulated both in WT and *Tyk2*^−/−^ cultures with an additive response more noticeable with CL097 than with IMIQ (**P versus *P, Table S6). Intriguingly, the responses of WT and *Tyk2*^−/−^ B cells were comparable (Fig. 5B and Table S6). The addition of IFNα to the different TLR7 agonists also led to greater plasmablast differentiation (Fig. 5D and Table S6), mostly in WT cultures, which presented higher CD138^+^ cell percentages than *Tyk2*^−/−^ B cell cultures stimulated with IMIQ + IFNα. Therefore, there is an additive in vitro proliferative and differentiation response of IFNα with TLR7L, which is larger with CL097 than with IMIQ and partially compensates for the absence of TYK2.

### Impaired TLR7 expression, cytokine and antibody secretion of *Tyk2*^−/−^ splenic B cells in response to TLR7 ligands.

**Fig. 6:**
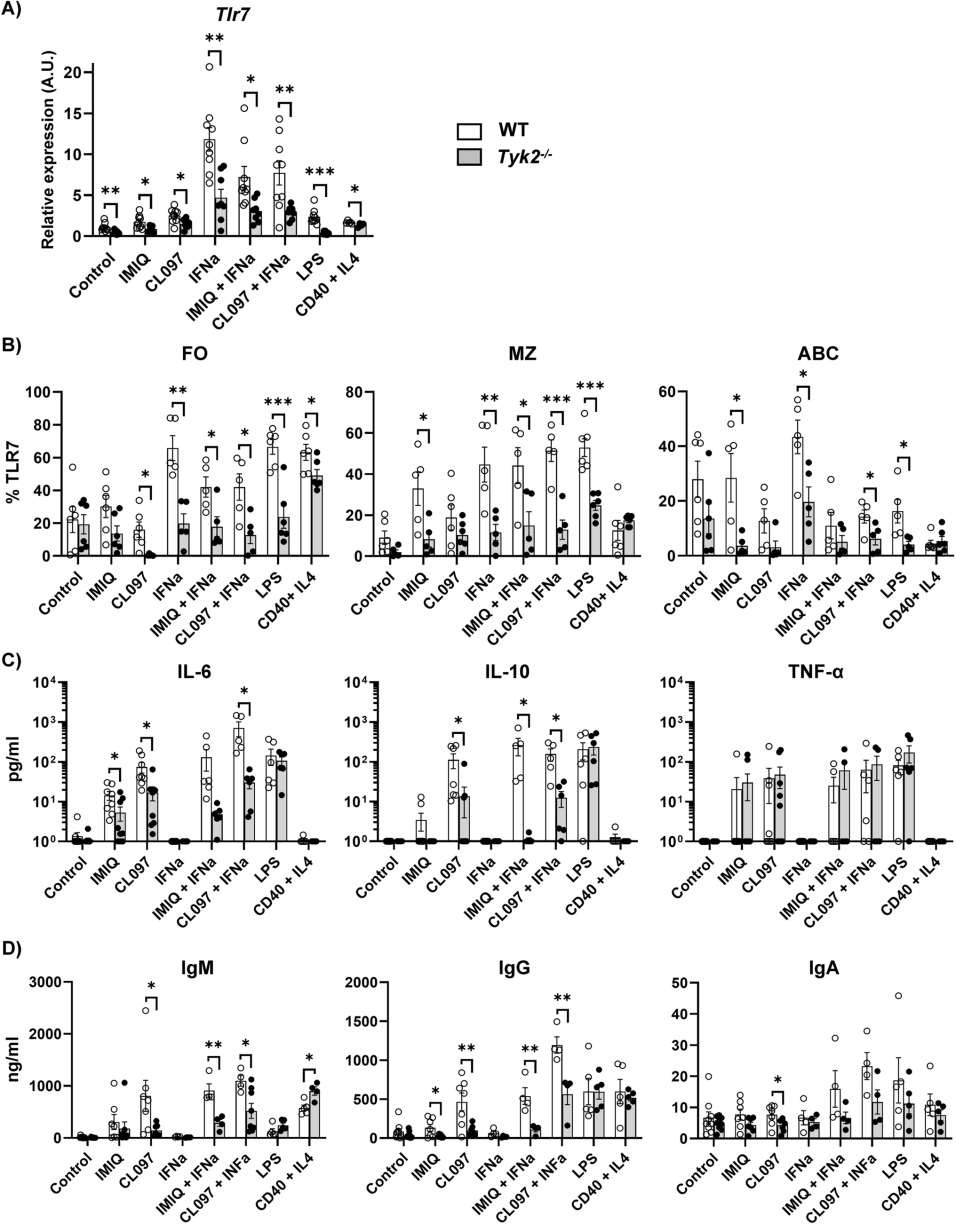
TLR7 expression, cytokine and humoral response in splenic B cell cultures. After 6 h, 72 h or 96 h in culture, cells were recovered and used for qPCR or were stained with anti-TLR7, anti-CD21 and anti-CD23, and the supernatants were used to quantify their cytokine or Ig content. **A** qPCR of *Tlr7* at 6 h. RNA was extracted from samples from B cell cultures and subjected to TLR7-specific qPCR. **B** Cells obtained after 72 h in culture were stained as in Fig. 4B to determine the TLR7 intracellular content in the FO, MZ and ABC subpopulations developed in the cultures. Data are shown as relative numbers (%) of TLR7^+^ cells (referred to the corresponding isotype control). **C** Supernatants obtained from B cell cultures at 72 h were subjected to cytometric bead arrays (CBA) to determine their IL-6, IL-10 and TNF-α content. **D** Antibody secretion was measured by ELISA from cultures at 96 h. Results are presented as individual values (dots), the bars show the mean ± SEM (n = 4–6). Comparisons were performed by Student's t-test: * *P* < 0.05; ** *P* < 0.01; *** *P* < 0.001

Since TLR7L-induced B cell differentiation was diminished in *Tyk2*^*−/−*^ cells, we considered studying the expression of *Tlr7* and several genes involved in antibody-producing cell formation. For this purpose, cells from the stimulated cultures were obtained and RNA was prepared to evaluate early (6 h) and late (48 h) gene response by qPCR (Fig. 6A and Fig. S3). *Tlr7* expression was diminished in untreated *Tyk2*^*−/−*^ compared to WT cells and was induced in response to all the treatments tested except for LPS in *Tyk2*^*−/−*^ cells (Fig. 6A and Table S7). However, *Tyk2*^*−/−*^ B cells failed to match the levels of WT B cells in response to the stimuli. According to these data, TLR7 protein expression (measured by intracellular flow cytometry) in gated FO, MZ and ABC developing in these cultures was also lower in *Tyk2*^*−/−*^ compared to WT controls (Fig. 6B). Addition of TLR7 ligands alone did not induce *Stat1* (Fig. S3). IFNα up-regulated *Stat1* expression, both in WT and *Tyk2*^−/−^ cells, although in the latter the induction was significantly lower. This difference was maintained in the presence of TLR7L + IFNα. Interestingly, *Ifnar1* and *Jak1* showed no changes in response to TLR7L or IFNα stimulation (Fig. S3). Of note, LPS scarcely induced *Stat1* and *Tlr7* expression in *Tyk2*^−/−^cells (see Fig. S3 and Fig. 6A), supporting the idea of a downregulated inflammation leading to the lack of sepsis described in *Tyk2*^−/−^ mice in response to LPS [35, 36]. Regarding transcripts related to plasmablast differentiation such as B-lymphocyte-induced maturation protein-1 (*Blimp1*), X-Box Binding Protein 1 (*Xbp1*), or Activation-Induced Cytidine Deaminase (*Aicd*) genes [60–62], they were induced by TLR7L regardless of the presence or absence of IFN (Fig. S3).

Secreted cytokines were measured by cytometric bead array in the supernatants of splenic B cell cultures at 72 h (Fig. 6C). Of the 13 cytokines tested in the inflammation panel (see Material and methods), IL-6, IL-10 and TNF-α were detected in the supernatants. Production of IL-6 by B cells increases the pro-inflammatory status in response to infections or in autoimmune pathologies [63]. Furthermore, IL10-producing B cells are related with the presence of regulatory B cells (CD1d^+^CD5^+^), which may be present in the pool of splenic B cells used in the cultures [64]. We showed that IL-6 and IL-10 levels maintained a pattern that paralleled those observed in differentiation and proliferation under TLR7L stimulation. In particular, *Tyk2*^−/−^ B cells produced lower levels of IL-6 compared to WT B cells in response to IMIQ and CL097. The addition of IFNα to CL097 had an additive effect in WT B cells, but this was not the case in *Tyk2*^−/−^ cells (*P versus ns, Table S8). By contrast, no differences were detected in the other conditions tested (Fig. 6C). These effects were alike for the secretion of IL-10, whereas TNF-α production showed no differences in *Tyk2*^−/−^ and WT B cells in response to TLR7L (Fig. 6C).

The secretion of IgM and class-switched IgA and IgG from cultured splenic B cells was assessed by ELISA after 96 h of culture with TLR7L (Fig. 6D). In response to LPS the Ig levels detected were similar. However, when stimulated with TLR7L, IgM and IgG, secretion was lower in *Tyk2*^−/−^ B cells compared to controls. The IFNα had an additive effect with TLR7L, more evident with CL097 compared to IMIQ. This effect was observed both in WT and in *Tyk2*^−/−^ B cells, although the latter secreted lower levels than WT B cells. Also, IgA levels were lower in *Tyk2*^−/−^ B cells stimulated with CL097 and there was an additive response induced by the addition of IFNα in WT B cells. This effect was less evident in *Tyk2*^−/−^ B cells, in which little or no IgA induction was observed relative to untreated B cells. Overall, the results showed that *Tyk2*^−/−^ splenic B cells exhibited a diminished humoral response. From these results we conclude that *Tyk2* deficiency decreases TLR7 expression and affects the responses through this receptor. This impaired TLR7 expression seems to be caused by a diminished type I IFN feedback loop and affects B cells.

## Discussion

Type I IFNs are crucial in the antiviral response and modulation of the immune system. They act through IFNAR, which binds the cytoplasmic tyrosine kinases JAK1 and TYK2 initiating the IFN-I signaling pathway. TYK2 loss-of-function variants have been described as protective in the outcome of autoimmune diseases [25, 26]). Conversely, overexpression of TYK2 and certain proteins of its signaling pathway is associated with increased severity in the SARS-CoV-2 response [24].

Previously, our group reported in patients with B cell acute lymphoblastic leukemia TYK2 variants that impaired the response to IFNα in vitro [27]. Therefore, we sought to ascertain the effect of TYK2 deficiency on B cell populations in *Tyk2*^*−/−*^ mice. Our results show that, while B cell populations in the bone marrow show no apparent alterations, in agreement with previous data [40, 41], splenic B cell populations exhibit remarkable changes under basal conditions, with a decrease of MZ B cells and an augmentation of ABC, while FO B cells remain unchanged. Previous analyses using the total number of splenic nucleated cells had overlooked these significant differences [41]. Other genetically modified mice in which MZ cells have been found to be decreased are *Tlr8*^*−/−*^ and *Tlr8/Tlr9*^*−/−*^ mice [65]. On the other hand, MZ B cell precursors (CD21^hi^IgM^hi^CD23^hi^) are increased in the autoimmune BXD2 mouse strain, which exhibits high levels of IFNα [66] underlining the central role of IFNα in the regulation of MZ B cells.

RNA-seq data comparing MZ and FO B cells from WT and *Tyk2*^−/−^ mice under homeostatic conditions showed a signature of down-regulated IFN-I responsive genes that included programmed cell death genes, shared by MZ and FO B cells. TYK2 deficiency decreased gene expression related to inflammatory necroptosis and pyroptosis cell death signaling pathways in FO and MZ cells. In the last years, novel regulators such as the innate immune sensor Z-DNA binding protein (ZBP1) and the crosstalk between pyroptosis, apoptosis and necroptosis have been characterized [67]. IFN-I-mediated apoptosis in bone marrow-derived adult B cells and pro-B cells and the activation of CASP11-induced pyroptosis in mice is dependent on TYK2 [36, 40–42]. Our data also support the involvement of TYK2 in ZBP1, CASP1 and CASP4 regulation in splenic B cells, although the specific requirements and the understanding of these pathways deserve further investigation. In addition, there were also specific DEGs in each type of cell. In this regard, the canonical gene pathway that was most affected in MZ but not in FO B cells was EIF2 signaling [68]. The translation factor EIF2 is crucial for translation initiation and, therefore, reinforces the importance in MZ B cells of the type I IFN/TYK2 axis in protein synthesis and stress responses against viruses [69].

Pattern recognition receptors (PRRs) activate signaling cascades that lead to transcription of IFN-I, which triggers IFN-I responses through the IFNAR/JAK axis [70]. IFN-stimulated genes encode a network of intermediate proteins that modify the response by feedforward and feedback regulation of the IFN signaling pathway [71, 72]. We observed that under homeostasis, in the absence of TYK2, poor IFNα-stimulated gene expression was detected in MZ and FO B cells and, among PRRs, only *Tlr7* was identified as a DEG. In consequence, *Tyk2*^−/−^ B cell splenic cultures showed weak proliferation and differentiation in response to TLR7L, while the response to TLR4 ligand or anti-CD40 + IL-4 was similar to that of WT B cells. Addition of IFNα to the cultures, alone or plus TLR7L, induced TLR7 expression. These data support previous reports on induction of TLR7 expression in B cells [73, 74], although in our case the low basal TLR7 expression in *Tyk2*^*−/−*^ splenic B cells precluded reaching WT expression after B cell stimulation, both at the RNA and protein level. These lower TLR7 levels seem to be enough to trigger normal B cell proliferation, suggesting a complete activation of the IFNAR/JAK axis via JAK1 signaling [29, 75]. However, neither the differentiation nor the humoral response matched that of WT B cells, even in the presence of IFNα plus TLR7L. Therefore, TYK2 seems to specifically mediate the complete differentiation to antibody-producing cells mediating Ig and cytokine responses.

In vitro B cell stimulation with anti-CD40 + IL-4 mimics the stimulation triggered by the interaction of FO B cells with T follicular helper (Tfh) cells through the BCR, promoting B cell differentiation and humoral responses [45, 76]. In our model, splenic *Tyk2*^−/−^ B cells did not release IL-6, IL-10 or TNF-α in response to anti-CD40 + IL-4. In addition, although upregulated IgM production was detected in *Tyk2*^−/−^ B cells compared to WT cells, no differences were observed in IgG or IgA secretion. In humans with TYK2 loss-of-function mutations, polarization toward Th2 cytokine production increases in memory CD4^+^ T cells, whereas Th17 cytokines and IL-10 secretion decreases [26, 39]. The generation of memory B cells is not affected in these patients, while there is a trend for more switched memory B cells [39]. However, healthy individuals homozygous for the protective autoimmune TYK2 P1104A variant exhibit a decrease in both Tfh cells and switched memory B cells [26]. However, studies on a murine model homozygous for TYK2 P1104A showed no impact on Tfh and germinal center B cell formation following T-dependent immunization [26]. Further investigation of T-dependent responses in *Tyk2*^−/−^ mice will elucidate whether germinal center reaction is affected by TYK2 deficiency.

**Fig. 7:**
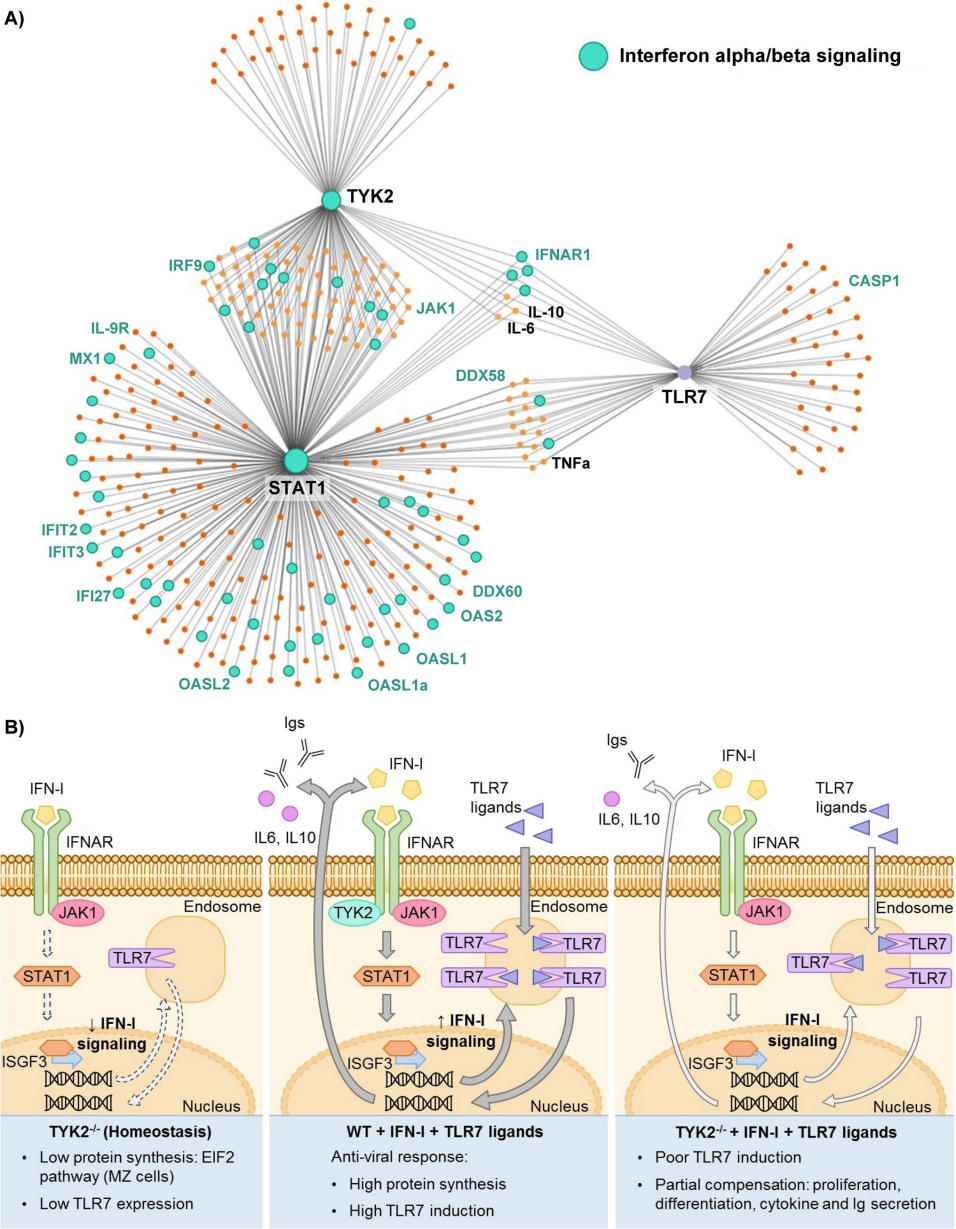
**A** Gene network crosstalk between selected DEGs (TYK2, STAT1 and TLR7) created with NetworkAnalyst software [44]. Predicted protein–protein interactions involved in the canonical IFNα/β pathway are colored in turquoise. Named proteins are those found disrupted as consequence of TYK2 deficiency; in turquoise appear those shared both by MZ and by FO B cells analyzed by RNA-seq (17 out of a total of 49) or by qPCR (JAK1); in black are those analyzed by cytometry (IFNAR1) or ELISA (IL6, IL10, TNF-α). **B** Proposed model of the orchestration of IFN-I and TLR7 signaling by TYK2 in splenic B cells. Left panel: *Tyk2*^−/−^ cells in homeostasis showed down-regulation of IFN-I signaling pathway and low TLR7 expression. Middle panel: in WT cells, in the presence of IFN-I and TLR7L forward IFN-I signaling through IFNAR induces TLR7 expression which in turn, upon binding to its ligands induces IFN-I, Igs and cytokine secretion. Right panel: upon activation with IFN-I plus TLR7L, *Tyk2*^−/−^ cells poorly induce TLR7, are unable to proliferate and differentiate like WT cells and secrete lower amounts of cytokines and antibodies. TLR7 is a target of the IFN-I pathway in splenic B cells

In addition to *TLR7*, other IFN-regulated *TLRs* have been described (*i.e.*, *TLR1*, *TLR3, TLR5* and *TLR8*) [77]. The IFN-regulated *TLRs* exhibit enrichment of conserved IFN-responsive elements (ISRE) and STAT1 sites on their promoters. Interestingly, the TLR7 promoter has four putative conserved sites for STAT1 [78] suggesting a fine tuning of TLR7 expression by this transcription factor. Consistent with this, we have found that *Stat1* is the only member of the STAT family differentially downregulated in *Tyk2*^−/−^ MZ and FO B cells. These data agree with the low STAT1 expression found in bone marrow-derived macrophages of *Tyk2*^−/−^ mice [28] and support the proposed role for STAT1 in MZ B cell differentiation [79]. Therefore, STAT1 seems to be a mediator in the crosstalk between TYK2 and TLR7 in activated cells, although the involvement of other genes cannot be ruled out (Fig. 7A). We propose that, in *Tyk2*^*−/−*^ MZ and FO B cells, the tonic signal proposed to be induced by constitutive type I IFN without stimulus [71] is very low, if exists, thereby precluding the TLR7 activation necessary for optimal differentiation and proliferation (Fig. 7B).

Our data reveal that TYK2 has an impact on TLR7 expression and function, and this crosstalk is mediated by type I IFN signaling. The activation of this gene network influences B cell distribution, the differentiation and the establishment of MZ B cells as well as the production of cytokines and Igs. In addition, this mechanism may explain the greater susceptibility to viruses and encapsulated bacteria found in *Tyk2*-deficient patients [19–21, 23], and in *Tyk2*^−/−^ mice [28]. Furthermore, *Tyk2* deficiency protects mice from severe inflammation in Systemic Lupus Erythematosus (SLE), arthritis and psoriasis [31, 33, 34]. In fact, a tight control and regulation of TLR7 is pivotal for avoiding SLE and inflammatory pathology in mice [65]. Therefore, we provide compelling evidence that TLR7 is a specific target of a TYK2 orchestrated IFN-I mediated feedback loop in B cells. Consequently, the possible therapeutic use of TYK2 inhibitors in autoimmune diseases should take into account their impact on TLR7 expression, which may lead to a low antiviral response in these patients. Similarly, the use of TLR7-activating adjuvants in viral vaccines may be useful for patients with TYK2 loss-of-function variants.

### Supplementary Information

Below is the link to the electronic supplementary material.

Supplementary file1 (PDF 1879 KB)

## Data Availability

The datasets generated during the current study are included in the Supplementary Material. Further inquiries can be directed to the corresponding author.

## Abbreviations

TYK2: Tyrosine kinase 2
*Tyk2*^−/−^: TYK2-deficient mice
ABC: Aged B cells
MZ: Marginal zone
FO: Follicular
TLR7: Toll-like receptor 7
TLR7L: Toll-like receptor 7 ligands
IFN-I: Type I interferon
IFNAR: IFN α/β receptor
DEG: Differentially expressed gene
WT: Wild type
Igs: Immunoglobulins
LPS: Lipopolysaccharide
