# Correction: Tyrosine kinase 2 modulates splenic B cells through type I IFN and TLR7 signaling

**DOI:** 10.1007/s00018-024-05482-y

**Published:** 2024-11-15

**Authors:** Irene Bodega-Mayor, Pablo Delgado-Wicke, Alejandro Arrabal, Estíbaliz Alegría-Carrasco, Ana Nicolao-Gómez, Marta Jaén-Castaño, Cristina Espadas, Ana Dopazo, Enrique Martín-Gayo, María Luisa Gaspar, Belén de Andrés, Elena Fernández-Ruiz

**Affiliations:** 1 Molecular Biology Unit, Hospital Universitario de La Princesa and Research Institute (IIS-Princesa), Madrid, Spain; 2 Present Address: Immunobiology Unit, Centro Nacional de Microbiología, Instituto de Salud Carlos III, Majadahonda, Madrid, Spain; 3 Genomics Unit, Centro Nacional de Investigaciones Cardiovasculares, Madrid, Spain; 4 CIBER de Enfermedades Cardiovasculares (CIBERCV), Madrid, Spain; 5 Immunology Department, Hospital Universitario de La Princesa and IIS-Princesa, Madrid, Spain; 6 Faculty of Medicine, Universidad Autónoma de Madrid, Madrid, Spain

**Correction: Cellular and Molecular Life Sciences (2024) 81:199** 10.1007/s00018-024-05234-y

In the published article, the authors would like to update the below mentioned three errors.

Enrique Vázquez de Luis at affiliation ‘Genomics Unit, Centro Nacional de Investigaciones Cardiovasculares, Madrid, Spain’ was missing from the author list and in the author contribution section: All authors contributed to the study conception and design. Material preparation, data collection and analysis were performed by Irene Bodega-Mayor, Pablo Delgado Wicke, Alejandro Arrabal, Estíbaliz Alegría-Carrasco, Ana Nicolao-Gómez and Marta Jaén-Castaño. Transcriptomic experiments and analysis: Cristina Espadas, Ana Dopazo, Enrique Vázquez de Luis and Enrique Martín-Gayo. The first draft of the manuscript was written by Elena Fernández-Ruiz and Belén de Andrés, and all authors commented on previous versions of the manuscript. All authors read and approved the final manuscript.

In Fig. 3b of this article, the values used to depict the volcano plot for MZ cells do not correspond to the MZ data included in Supplementary Tables S4 and S5. The correct MZ data is shown below, although no significant changes were detected on the final Fig. 3.

**Fig. 3:**
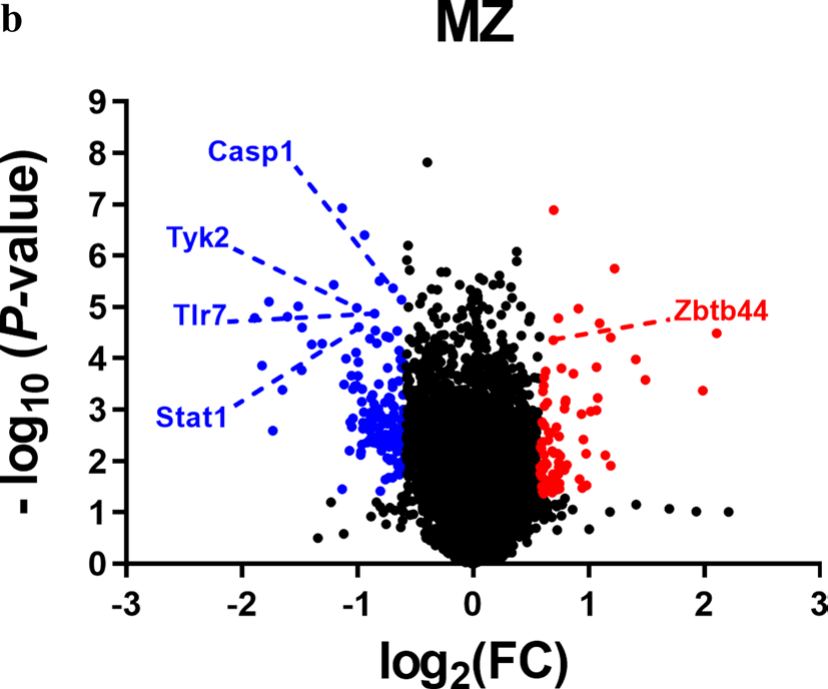
RNA-Seq analysis of gene expression in FO and MZ B cells in homeostasis. FACS-purified FO and MZ cells from WT and *Tyk2*^−/−^ mice were used to prepare total RNA (MZ cells) or mRNA (FO cells) as indicated in the Material and Methods section. **B** Distribution of DEGs according to FO or MZ cell population plotted as a function of their variation and significance (*P*-value < 0.05). Labeled in red and blue are representative up- and down-regulated genes, respectively.

In Supplementary Table 3 of this article there are some errors in the log_2_(FC) values for FO cells, which did not correspond to the values used to depict the heat map and volcano plot of the FO cells shown in Fig. 3. These values have been corrected in the updated Supplementary Table 3.

The original article has been corrected.

## Supplementary Information

Below is the link to the electronic supplementary material.

Supplementary file1 (DOCX 22 KB)

