# Supplementary material for: Correction: Tyrosine kinase 2 modulates splenic B cells through type I IFN and TLR7 signaling
Source: Cell Mol Life Sci. 2024 Nov 15;81(1):455. doi: 10.1007/s00018-024-05482-y (PMC11568076; doi:10.1007/s00018-024-05482-y)
Supplement: Supplementary file 1 — Supplementary file1 (DOCX 22 KB) [file 18_2024_5482_MOESM1_ESM.docx]

**Table S3.** Differentially expressed genes (DEGs) of follicular (FO) cells between WT and *Tyk2^-/-^* mice and their respective fold-changes (FC), *P*-values and false discovery rate (FDR).

| **Transcript ID** | **Gene name** | **log_2_(WT_FO)** | **log_2_(KO_FO)** | **log_2_(FC)** | ***P*-value** | **FDR < 0.05** |
| --- | --- | --- | --- | --- | --- | --- |
| ENSMUSG00000020279 | Il9r | 2.856165659 | 1.451681938 | -1.404483721 | 3.10286E-08 | 0.00026167 |
| ENSMUSG00000041827 | Oasl1 | 1.218996298 | 0.102168095 | -1.116828203 | 4.56E-08 | 0.00026167 |
| ENSMUSG00000032175 | Tyk2 | 2.623989179 | 1.179992767 | -1.443996413 | 1.05E-07 | 0.000453443 |
| ENSMUSG00000033538 | Casp4 | 1.220044568 | 0.431459816 | -0.788584752 | 1.32307E-07 | 0.000455559 |
| ENSMUSG00000025498 | Irf7 | 1.689214747 | 0.706201115 | -0.983013632 | 4.9925E-07 | 0.001432516 |
| ENSMUSG00000073491 | Ifi213 | 2.804907512 | 0.917339091 | -1.887568421 | 3.79065E-06 | 0.005321084 |
| ENSMUSG00000064215 | Ifi27 | 1.605589822 | 0.46764948 | -1.137940342 | 4.02E-06 | 0.005321084 |
| ENSMUSG00000017830 | Dhx58 | 1.123316612 | 0.392857324 | -0.730459288 | 4.33E-06 | 0.005321084 |
| ENSMUSG00000096255 | Dynlt1b | 0.376155062 | 1.288137549 | 0.911982487 | 7.14E-06 | 0.007683403 |
| ENSMUSG00000070327 | Rnf213 | 4.569050624 | 3.395006163 | -1.174044461 | 7.69E-06 | 0.007783618 |
| ENSMUSG00000054404 | Slfn5 | 2.464472562 | 0.406666293 | -2.057806269 | 8.36E-06 | 0.007998596 |
| ENSMUSG00000032661 | Oas3 | 0.831296603 | 0.029338081 | -0.801958521 | 1.02789E-05 | 0.00888429 |
| ENSMUSG00000037921 | Ddx60 | 0.739247108 | 0.034030153 | -0.705216956 | 1.0321E-05 | 0.00888429 |
| ENSMUSG00000044583 | Tlr7 | 1.462124564 | 0.508475146 | -0.953649418 | 1.47899E-05 | 0.010123034 |
| ENSMUSG00000079017 | Ifi27l2a | 1.633679872 | 0.135904454 | -1.497775418 | 1.53E-05 | 0.010123034 |
| ENSMUSG00000110537 | Gm4316 | 3.078176783 | 4.130420518 | 1.052243734 | 1.86E-05 | 0.011356231 |
| ENSMUSG00000025743 | Sdc3 | 0.649098334 | 0.011609386 | -0.637488948 | 2.10E-05 | 0.011356231 |
| ENSMUSG00000057596 | Trim30d | 1.683545313 | 1.014707115 | -0.668838198 | 2.29E-05 | 0.011919874 |
| ENSMUSG00000035208 | Slfn8 | 2.455872719 | 1.319837156 | -1.136035563 | 4.85E-05 | 0.019531135 |
| ENSMUSG00000073409 | H2-Q6 | 5.537036705 | 4.595062665 | -0.94197404 | 5.07433E-05 | 0.019806071 |
| ENSMUSG00000034422 | Parp14 | 3.213749187 | 2.473788968 | -0.739960219 | 5.17701E-05 | 0.019806071 |
| ENSMUSG00000033880 | Lgals3bp | 0.901196264 | 0.181128308 | -0.720067956 | 5.68361E-05 | 0.020385203 |
| ENSMUSG00000032690 | Oas2 | 1.135938145 | 0.008130826 | -1.127807319 | 5.86E-05 | 0.020598633 |
| ENSMUSG00000029322 | Plac8 | 2.519536613 | 1.785234894 | -0.734301719 | 6.85E-05 | 0.021944988 |
| ENSMUSG00000029561 | Oasl2 | 0.825419678 | 0.095363992 | -0.730055686 | 6.8833E-05 | 0.021944988 |
| ENSMUSG00000097893 | 1700034P13Rik | 1.430837831 | 2.4393409 | 1.008503069 | 9.40E-05 | 0.02742817 |
| ENSMUSG00000075602 | Ly6a | 2.635366901 | 0.756047518 | -1.879319384 | 0.000100242 | 0.027834991 |
| ENSMUSG00000105263 | Gm42427 | 4.560179669 | 6.12685471 | 1.56667504 | 0.000103684 | 0.02791418 |
| ENSMUSG00000061232 | H2-K1 | 6.676278625 | 6.012636057 | -0.663642568 | 0.00010377 | 0.02791418 |
| ENSMUSG00000096780 | Tmem181b-ps | 0.121501003 | 0.721974762 | 0.600473759 | 0.000108961 | 0.028859475 |
| ENSMUSG00000020282 | Rhbdf1 | 1.274734689 | 0.570829498 | -0.703905191 | 0.000113132 | 0.029510302 |
| ENSMUSG00000078606 | Gm4070 | 3.810026408 | 2.086522061 | -1.723504347 | 0.000127761 | 0.031843911 |
| ENSMUSG00000105703 | Gm43305 | 6.868643871 | 8.039183996 | 1.170540125 | 0.00014852 | 0.03502634 |
| ENSMUSG00000110386 | Gm42031 | 4.577435121 | 6.046192628 | 1.468757507 | 0.000218941 | 0.040970564 |
| ENSMUSG00000000386 | Mx1 | 0.76659608 | 0.004686865 | -0.761909215 | 0.000229679 | 0.042517756 |
| ENSMUSG00000052776 | Oas1a | 0.700977229 | 0.00730421 | -0.693673019 | 0.000236333 | 0.042828599 |
| ENSMUSG00000074896 | Ifit3 | 0.659887783 | 0.02149299 | -0.638394793 | 0.000264124 | 0.045021421 |
| **Transcript ID** | **Gene name** | **log_2_(WT_FO)** | **log_2_(KO_FO)** | **log_2_(FC)** | ***P*-value** | **FDR < 0.05** |
| ENSMUSG00000046841 | Ckap4 | 1.539013091 | 2.319639171 | 0.78062608 | 0.000317226 | 0.049648734 |
| ENSMUSG00000095041 | AC149090.1 | 4.36033084 | 5.374407266 | 1.014076426 | 0.000377501 | 0.055076702 |
| ENSMUSG00000037849 | Ifi206 | 2.364958347 | 1.413761481 | -0.951196866 | 0.000424255 | 0.058876016 |
| ENSMUSG00000103865 | Gm37416 | 0.780847554 | 1.514876868 | 0.734029315 | 0.0004309 | 0.058876016 |
| ENSMUSG00000002325 | Irf9 | 2.712355952 | 1.996378357 | -0.715977595 | 4.44E-04 | 0.059320019 |
| ENSMUSG00000082286 | Pisd-ps1 | 2.414403999 | 3.143845633 | 0.729441634 | 0.000499973 | 0.064235347 |
| ENSMUSG00000053835 | H2-T24 | 1.732565624 | 1.151041391 | -0.581524233 | 0.000543416 | 0.066350747 |
| ENSMUSG00000097971 | Gm26917 | 4.387933869 | 3.438252295 | -0.949681574 | 6.81E-04 | 0.075438876 |
| ENSMUSG00000030921 | Trim30a | 3.066419399 | 2.18736237 | -0.87905703 | 0.000711356 | 0.076449826 |
| ENSMUSG00000105373 | Gm42429 | 1.830246612 | 2.819201014 | 0.988954402 | 0.000749143 | 0.078552077 |
| ENSMUSG00000027514 | Zbp1 | 1.387453979 | 0.705883847 | -0.681570132 | 0.000778516 | 0.080055316 |
| ENSMUSG00000060550 | H2-Q7 | 5.699833288 | 4.738023322 | -0.961809966 | 7.81E-04 | 0.080055316 |
| ENSMUSG00000045932 | Ifit2 | 0.844754795 | 0.247377378 | -0.597377417 | 0.000842863 | 0.084264219 |
| ENSMUSG00000105987 | AI506816 | 3.053500797 | 3.643693602 | 0.590192805 | 0.000988124 | 0.09138085 |
| ENSMUSG00000055413 | H2-Q5 | 3.992485497 | 3.319222432 | -0.673263065 | 0.00116141 | 0.098964304 |
| ENSMUSG00000104713 | Gbp6 | 0.826094321 | 0.089116145 | -0.736978176 | 1.48E-03 | 0.113094002 |
| ENSMUSG00000035929 | H2-Q4 | 4.780985374 | 4.197649806 | -0.583335568 | 0.001517543 | 0.11428119 |
| ENSMUSG00000072620 | Slfn2 | 2.978663766 | 2.177903301 | -0.800760465 | 1.52E-03 | 0.11428119 |
| ENSMUSG00000025888 | Casp1 | 1.562455432 | 0.947565774 | -0.614889658 | 0.001644965 | 0.119492462 |
| ENSMUSG00000066677 | Ifi208 | 1.927322726 | 1.121185715 | -0.806137011 | 1.80E-03 | 0.12660178 |
| ENSMUSG00000046879 | Irgm1 | 1.761383298 | 1.163271712 | -0.598111585 | 0.00233851 | 0.143784929 |
| ENSMUSG00000096768 | Erdr1 | 3.057707749 | 4.655351121 | 1.597643372 | 0.003022915 | 0.166269988 |
| ENSMUSG00000074151 | Nlrc5 | 3.06153918 | 2.397022386 | -0.664516794 | 0.003304603 | 0.172731945 |
| ENSMUSG00000054364 | Rhob | 4.717439843 | 4.123527247 | -0.593912596 | 0.004009438 | 0.193622962 |
| ENSMUSG00000062783 | Csprs | 1.294398136 | 0.573038718 | -0.721359419 | 4.60E-03 | 0.205796018 |
| ENSMUSG00000111202 | AC153954.3 | 2.079266159 | 2.974443441 | 0.895177283 | 0.004957461 | 0.208925782 |
| ENSMUSG00000026104 | Stat1 | 3.663134664 | 2.902057526 | -0.761077138 | 4.97E-03 | 0.208925782 |
| ENSMUSG00000095134 | Mid1-ps1 | 1.358845826 | 0.089041825 | -1.269804001 | 0.005337119 | 0.215542357 |
| ENSMUSG00000078921 | Tgtp2 | 0.980352131 | 0.354522206 | -0.625829925 | 0.005716327 | 0.219815738 |
| ENSMUSG00000036181 | Hist1h1c | 2.816098867 | 2.124182411 | -0.691916456 | 0.006769416 | 0.233433153 |
| ENSMUSG00000035202 | Lars2 | 4.775945175 | 3.552642544 | -1.223302631 | 6.95E-03 | 0.233433153 |
| ENSMUSG00000056116 | H2-T22 | 2.786547685 | 2.197905158 | -0.588642527 | 0.00741022 | 0.240253008 |
| ENSMUSG00000095562 | Gm21887 | 0.426005803 | 1.427488758 | 1.001482955 | 0.008575855 | 0.24802043 |
| ENSMUSG00000070501 | Ifi214 | 1.695220537 | 1.10531976 | -0.589900777 | 0.00893012 | 0.248828807 |
| ENSMUSG00000024900 | Cpt1a | 2.996840061 | 2.366169501 | -0.630670559 | 0.00910955 | 0.24889219 |
| ENSMUSG00000102049 | Zbed6 | 0.675768237 | 0.095498645 | -0.580269592 | 0.01220133 | 0.274798325 |
| ENSMUSG00000098178 | Gm42418 | 6.933858241 | 6.098351065 | -0.835507176 | 0.013251235 | 0.286599569 |
| ENSMUSG00000039001 | Rps21 | 3.875291719 | 3.2721443 | -0.603147419 | 0.021548705 | 0.349324397 |
| ENSMUSG00000041481 | Serpina3g | 2.190829457 | 1.515371799 | -0.675457658 | 0.028949762 | 0.3881613 |
| ENSMUSG00000064341 | mt-Nd1 | 5.867906358 | 6.489006194 | 0.621099836 | 0.031003789 | 0.399317843 |
| ENSMUSG00000035299 | Mid1 | 3.432479898 | 1.781164912 | -1.651314986 | 3.43E-02 | 0.415980392 |
