# Supplementary material for: Tyrosine kinase 2 modulates splenic B cells through type I IFN and TLR7 signaling
Source: Cell Mol Life Sci. 2024 Apr 29;81(1):199. doi: 10.1007/s00018-024-05234-y (PMC11058799; doi:10.1007/s00018-024-05234-y)
Supplement: Supplementary file 1 [file 18_2024_5234_MOESM1_ESM.pdf]

Supplementary Information for:

Tyrosine kinase 2 modulates splenic B cells through type I IFN and TLR7 signaling

Irene Bodega-Mayor[1,*,†], Pablo Delgado-Wicke[1,†], Alejandro Arrabal[2], Estíbaliz Alegría-Carrasco[1], Ana Nicolao-Gómez[1], Marta Jaén-Castaño[1], Cristina Espadas[3], Ana Dopazo[3,4], Enrique Martín-Gayo[5,6], María Luisa Gaspar[2], Belén de Andrés[2,††], Elena Fernández-Ruiz[1,6,††]

[1]Molecular Biology Unit, Hospital Universitario de La Princesa and Research Institute (IIS-Princesa), Madrid, Spain.

[2]Immunobiology Unit, Centro Nacional de Microbiología, Instituto de Salud Carlos III, Majadahonda, Madrid, Spain.

[3]Genomics Unit, Centro Nacional de Investigaciones Cardiovasculares, Madrid, Spain.

[4]CIBER de Enfermedades Cardiovasculares (CIBERCV), Madrid, Spain.

[5]Immunology Department, Hospital Universitario de La Princesa and IIS-Princesa, Madrid, Spain.

[6]Faculty of Medicine, Universidad Autónoma de Madrid, Madrid, Spain.

*Current address: Immunobiology Unit, Centro Nacional de Microbiología, Instituto de Salud Carlos III, Majadahonda, Madrid, Spain.

[†]These authors contributed equally to this work and share first authorship.

[††]These authors contributed equally to this work and share senior authorship.

Corresponding author: Elena Fernández-Ruiz, efruiz@salud.madrid.org (ORCID: 0000-0001-5380-1686).

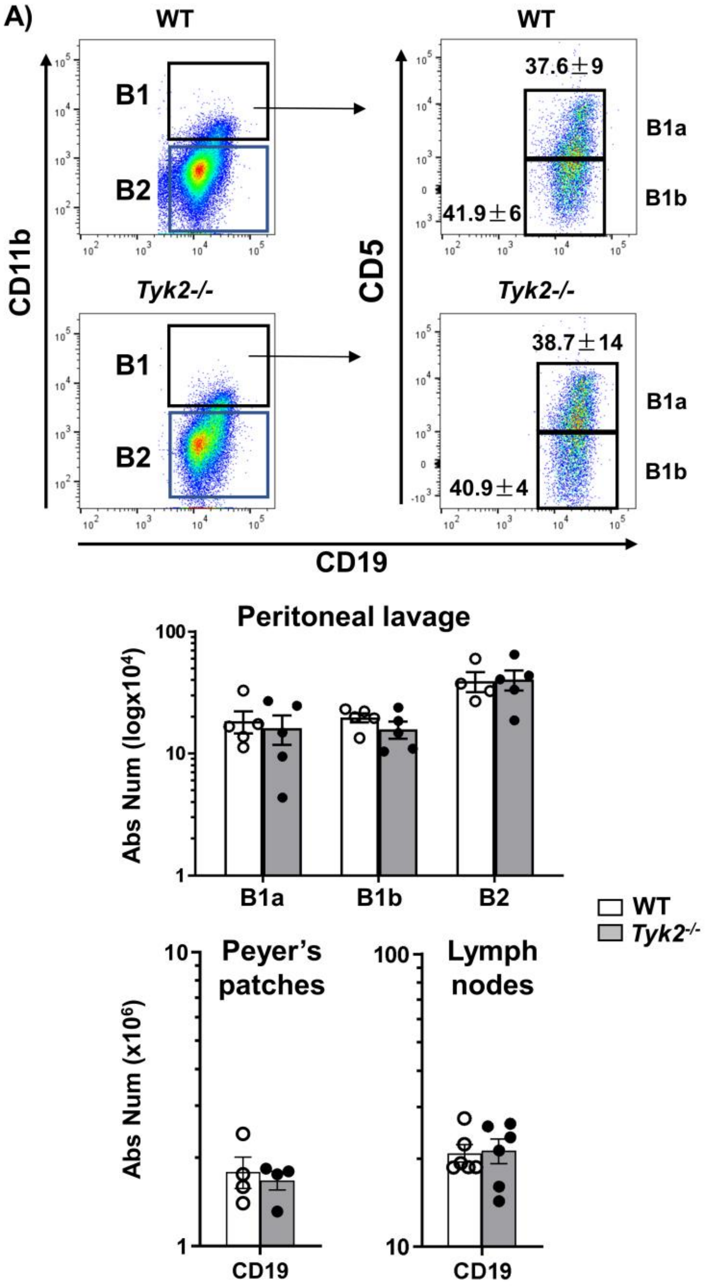

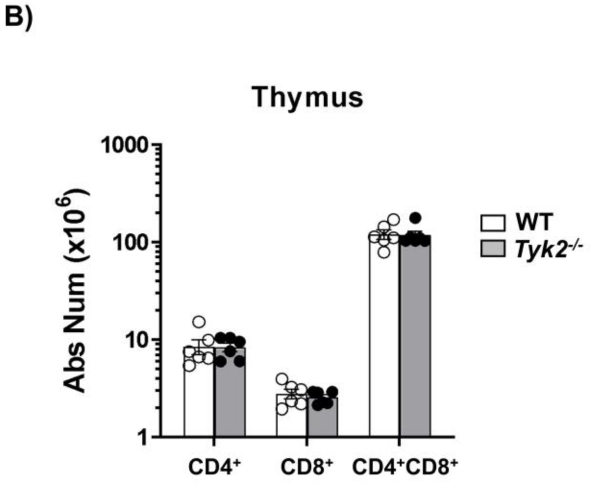

Fig. S1. Quantitation of B and T lymphocytes in different hematopoietic locations of WT and *Tyk2*$^{-/-}$ adult mice. A) B lymphocytes were analyzed in peritoneal lavages, Peyer's patches and lymph nodes. Upper left dot plots show a representative example of B2 cells (CD19^{+}CD11b^{-}) and B1 cells (CD19^{+}CD11b^{+}) and the discrimination between B1a and B1b on the basis of CD5 expression. Numbers denote percentage of each population expressed as means±SEM (n = 4-6). Lower left histograms show the quantitation of B cells on the peritoneal lavages, Peyer's patches and lymph nodes expressed as absolute numbers/sample. **B)** T lymphocytes in the thymus were quantitated using CD3, CD4 and CD8. Data represent means±SEM (n = 6).

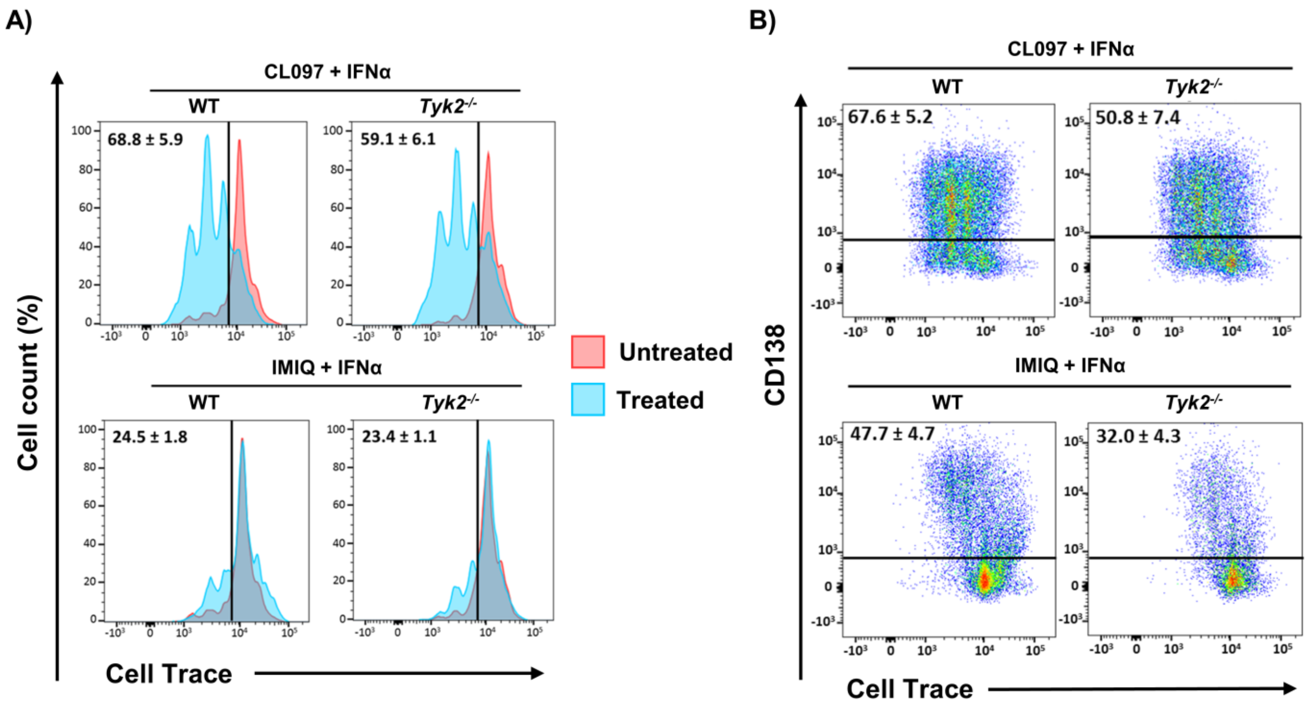

Fig. S2. Proliferation and differentiation of B cell cultures in the presence of IFNα. Splenic B cells were obtained as described in Materials and Methods and stained with Cell Trace at the beginning of the culture. **A)** Proliferation was analyzed in cultured cells treated with CL097 + IFNα or IMIQ + IFNα for 72 h. Overlaid histograms show a representative result for control cells (red) and treated cells (blue) in WT and *Tyk2*$^{-/-}$ mice. Percentage of proliferating cells (shown as numbers inside) was referred to control cells (black line). **B)** Differentiation of cultured B cells treated with CL097 + IFNα or IMIQ + IFNα for 72 h by the expression of CD138 and Cell Trace. Numbers denote percentage of CD138^{+} cells referred to isotype (black line).

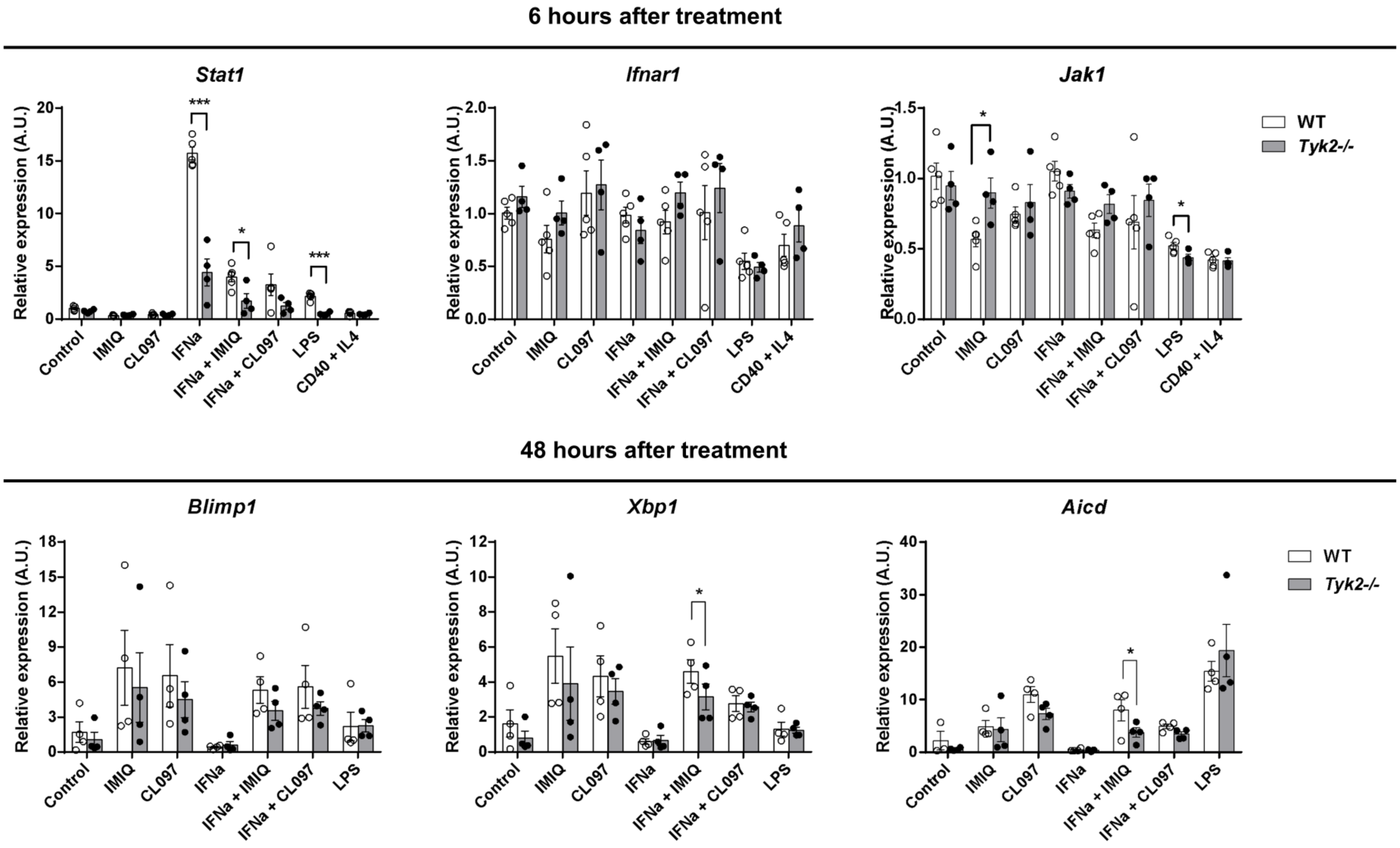

Fig. S3. **Expression of early and late response genes in splenic B cell cultures by qPCR.** Upper panels: *Stat1*, *Ifnar1* and *Jak1* were determined at 6 h. Cultures were set up as described in Materials and Methods. Lower panels: The B cell-differentiation related transcription factors *Blimp1*, *Xbp1* and *Aicd* were studied at 48 h of cell culture. RNA extraction procedures and qPCR reactions were performed as described in Fig. 4. Data are presented as relative expression arbitrary units (A.U.) using the $2^{-\Delta\Delta Ct}$ method (samples normalized to their *Gapdh* content). (n = 3-4 performed in triplicates). * $P < 0.05$; *** $P < 0.0001$.

Table S1. Antibodies employed in flow cytometry analysis.

MoAbs	Clone	Label	Company
B220	RA3-6B2	APC	BioLegend
CD3	17A2	PE-Cy7	BioLegend
CD4	GK1.5	APC	BioLegend
CD5	53-7.3	APC	BioLegend
CD8	53-5.8	PE	BioLegend
CD11b	M1/70	PE-Cy7, APC-Cy7	BioLegend
CD11c	N418	FITC	BioLegend
CD19	1D3	PE, BV421	eBioscience, BioLegend
CD21	7E9, 7G6	FITC, APC	BioLegend, BD Biosciences
CD23	B3B4	PE-Cy7	BioLegend
CD43	S11	PE	BioLegend
CD90.2	30-H12	Biotin	BioLegend
CD93	AA4.1	APC	BioLegend
CD138	281-2	APC	BioLegend
DX5	DX5	Biotin, APC	BioLegend
Gr-1	RB6-8C5	PE	BioLegend
IFNAR-1	MAR1-5A3	PE	BioLegend
IgM	RMM-1	PE	BioLegend
NK-1.1	S17016D	PE	BioLegend
T-bet	4B10	PE	BioLegend
TLR7	A94B10	PE	BioLegend
Isotype controls		FITC, PE, APC, PE-Cy7, APC-Cy7, BV421, BV510	BioLegend
LIVE/DEAD™		Fixable Aqua 405	Invitrogen
Propidium iodide		PI	Sigma Aldrich
Streptavidin		PE	BioLegend

Table S2. Phenotypes of the cell populations analyzed in this study.

Tissue	Population	Phenotype
**Spleen**	Natural killer T (NKT)	CD3^{+}DX5^{+}NK-1.1^{+}
	Granulocytes	CD11b^{+}GR1^{+}
	Monocytes	CD11b^{+}Gr-1^{-}
	Dendritic cells (DC)	Lin^{-} (Ly6G^{-}CD19^{-}DX5^{-}Thy-1.2^{-}) CD11c^{+}
	CD4^{+} T cells	CD3^{+}CD4^{+}CD8^{-}
	CD8^{+} T cells	CD3^{+}CD4^{-}CD8^{+}
	B cells	CD19^{+}
	Marginal zone B cells (MZ)	CD19^{+}CD21^{++}CD23^{lo}
	Follicular B cells (FO)	CD19^{+}CD21^{+}CD23^{++}
	Aged B cells (ABC)	CD19^{+}CD21^{lo}CD23^{lo} T-bet^{+}
	Hardy´s fractions A-C	B220^{+}CD43^{+}
	Hardy´s fractions D-F	B220^{+}CD43^{-}
	Transitionals T1	B220^{+}CD93^{+}IgM^{+}CD23^{-}
	Transitionals T2	B220^{+}CD93^{+}IgM^{+}CD23^{+}
	Transitionals T3	B220^{+}CD93^{+}IgM^{lo}CD23^{+}
**Peritoneal lavage**	B1a	CD19^{+}CD11b^{+}CD5^{+}
	B1b	CD19^{+}CD11b^{+}CD5^{-}
**Peyer's patches, lymph nodes**	B cells	CD19^{+}
	CD4^{+} T cells	CD3^{+}CD4^{+}CD8^{-}
	CD8^{+} T cells	CD3^{+}CD4^{-}CD8^{+}
	CD4^{+}CD8^{+} T cells	CD3^{+}CD4^{+}CD8^{+}

Table S3. Differentially expressed genes (DEGs) of follicular (FO) cells between WT and *Tyk2*$^{-/-}$ mice and their respective fold-changes (FC), *P*-values and false discovery rate (FDR).

Transcipt ID	Gene name	log_2(WT_FO)	log_2(KO_FO)	log_2(FC)	*P*-value	FDR < 0.05
ENSMUSG00000097893	1700034P13Rik	1.430837831	2.4393409	-1.116828203	4.55977E-08	0.00026167
ENSMUSG00000095041	AC149090.1	4.36033084	5.374407266	-1.404483721	3.10286E-08	0.00026167
ENSMUSG00000111202	AC153954.3	2.079266159	2.974443441	-1.443996413	1.05354E-07	0.000453443
ENSMUSG00000105987	AI506816	3.053500797	3.643693602	-0.788584752	1.32307E-07	0.000455559
ENSMUSG00000025888	Casp1	1.562455432	0.947565774	-0.983013632	4.9925E-07	0.001432516
ENSMUSG00000033538	Casp4	1.220044568	0.431459816	-0.730459288	4.32709E-06	0.005321084
ENSMUSG00000046841	Ckap4	1.539013091	2.319639171	-1.137940342	4.02264E-06	0.005321084
ENSMUSG00000024900	Cpt1a	2.996840061	2.366169501	-1.887568421	3.79065E-06	0.005321084
ENSMUSG00000062783	Csprs	1.294398136	0.573038718	0.911982487	7.14071E-06	0.007683403
ENSMUSG00000037921	Ddx60	0.739247108	0.034030153	-1.174044461	7.68596E-06	0.007783618
ENSMUSG00000017830	Dhx58	1.123316612	0.392857324	-2.057806269	8.36284E-06	0.007998596
ENSMUSG00000096255	Dynlt1b	0.376155062	1.288137549	-0.705216956	1.0321E-05	0.00888429
ENSMUSG00000096768	Erdr1	3.057707749	4.655351121	-0.801958521	1.02789E-05	0.00888429
ENSMUSG00000104713	Gbp6	0.826094321	0.089116145	-0.953649418	1.47899E-05	0.010123034
ENSMUSG00000095562	Gm21887	0.426005803	1.427488758	-1.497775418	1.5288E-05	0.010123034
ENSMUSG00000097971	Gm26917	4.387933869	3.438252295	1.052243734	1.86191E-05	0.011356231
ENSMUSG00000103865	Gm37416	0.780847554	1.514876868	-0.637488948	2.09748E-05	0.011356231
ENSMUSG00000078606	Gm4070	3.810026408	2.086522061	-0.668838197	2.28526E-05	0.011919874
ENSMUSG00000110386	Gm42031	4.577435121	6.046192628	-1.136035563	4.84913E-05	0.019531135
ENSMUSG00000098178	Gm42418	6.933858241	6.098351065	-0.739960219	5.17701E-05	0.019806071
ENSMUSG00000105263	Gm42427	4.560179669	6.12685471	-0.941974039	5.07433E-05	0.019806071
ENSMUSG00000105373	Gm42429	1.830246612	2.819201014	-0.720067956	5.68361E-05	0.020385203
ENSMUSG00000110537	Gm4316	3.078176783	4.130420518	-1.127807319	5.86276E-05	0.020598633
ENSMUSG00000105703	Gm43305	6.868643871	8.039183996	-0.730055686	6.8833E-05	0.021944988
ENSMUSG00000061232	H2-K1	6.676278625	6.012636057	-0.734301719	6.84626E-05	0.021944988
ENSMUSG00000035929	H2-Q4	4.780985374	4.197649806	1.008503069	9.39976E-05	0.02742817
ENSMUSG00000055413	H2-Q5	3.992485497	3.319222432	-1.879319384	0.000100242	0.027834991
ENSMUSG00000073409	H2-Q6	5.537036705	4.595062665	1.56667504	0.000103684	0.02791418
ENSMUSG00000060550	H2-Q7	5.699833288	4.738023322	-0.663642568	0.00010377	0.02791418
ENSMUSG00000056116	H2-T22	2.786547685	2.197905158	0.600473759	0.000108961	0.028859475
ENSMUSG00000053835	H2-T24	1.732565624	1.151041391	-0.703905191	0.000113132	0.029510302
ENSMUSG00000036181	Hist1h1c	2.816098867	2.124182411	-1.723504347	0.000127761	0.031843911
ENSMUSG00000037849	Ifi206	2.364958347	1.413761481	1.170540125	0.00014852	0.03502634
ENSMUSG00000066677	Ifi208	1.927322726	1.121185715	1.468757507	0.000218941	0.040970564
ENSMUSG00000073491	Ifi213	2.804907512	0.917339091	-0.761909215	0.000229679	0.042517756
ENSMUSG00000070501	Ifi214	1.695220537	1.10531976	-0.693673019	0.000236333	0.042828599
ENSMUSG00000064215	Ifi27	1.605589822	0.46764948	-0.638394793	0.000264124	0.045021421

Transcipt ID	Gene name	log_2(WT_FO)	log_2(KO_FO)	log_2(FC)	*P*-value	FDR < 0.05
ENSMUSG00000079017	Ifi27l2a	1,633679872	0,135904454	0,78062608	0,000317226	0,049648734
ENSMUSG00000045932	Ifit2	0,844754795	0,247377378	1,014076426	0,000377501	0,055076702
ENSMUSG00000074896	Ifit3	0,659887782	0,02149299	0,734029314	0,0004309	0,058876016
ENSMUSG00000020279	Il9r	2,856165659	1,451681938	-0,951196866	0,000424255	0,058876016
ENSMUSG00000025498	Irf7	1,689214747	0,706201115	-0,715977595	0,000444487	0,059320019
ENSMUSG00000002325	Irf9	2,712355952	1,996378357	0,729441634	0,000499973	0,064235347
ENSMUSG00000046879	Irgm1	1,761383298	1,163271712	-0,581524233	0,000543416	0,066350747
ENSMUSG00000035202	Lars2	4,775945175	3,552642544	-0,949681574	0,00068112	0,075438876
ENSMUSG00000033880	Lgals3bp	0,901196264	0,181128308	-0,87905703	0,000711356	0,076449826
ENSMUSG00000075602	Ly6a	2,635366901	0,756047517	0,988954402	0,000749143	0,078552077
ENSMUSG00000035299	Mid1	3,432479898	1,781164912	-0,681570132	0,000778516	0,080055316
ENSMUSG00000095134	Mid1-ps1	1.358845826	0.089041825	-0.961809966	0.000781209	0.080055316
ENSMUSG00000064341	mt-Nd1	5.867906358	6.489006194	-0.597377417	0.000842863	0.084264219
ENSMUSG00000000386	Mx1	0.76659608	0.004686865	0.590192805	0.000988124	0.09138085
ENSMUSG00000074151	Nlrc5	3.06153918	2.397022386	-0.673263065	0.00116141	0.098964304
ENSMUSG00000052776	Oas1a	0.700977229	0.00730421	-0.736978175	0.001484622	0.113094002
ENSMUSG00000032690	Oas2	1.135938145	0.008130826	-0.583335568	0.001517543	0.11428119
ENSMUSG00000032661	Oas3	0.831296603	0.029338081	-0.800760465	0.00152012	0.11428119
ENSMUSG00000041827	Oasl1	1.218996298	0.102168095	-0.614889658	0.001644965	0.119492461
ENSMUSG00000029561	Oasl2	0.825419678	0.095363992	-0.806137011	0.001801663	0.12660178
ENSMUSG00000034422	Parp14	3.213749187	2.473788968	-0.598111585	0.00233851	0.143784929
ENSMUSG00000082286	Pisd-ps1	2.414403999	3.143845633	1.597643372	0.003022915	0.166269988
ENSMUSG00000029322	Plac8	2.519536613	1.785234894	-0.664516794	0.003304603	0.172731945
ENSMUSG00000020282	Rhbdf1	1.274734689	0.570829498	-0.593912596	0.004009438	0.193622962
ENSMUSG00000054364	Rhob	4.717439843	4.123527247	-0.721359418	0.0046022	0.205796018
ENSMUSG00000070327	Rnf213	4.569050624	3.395006163	0.895177283	0.004957461	0.208925782
ENSMUSG00000039001	Rps21	3.875291719	3.2721443	-0.761077138	0.004970557	0.208925782
ENSMUSG00000025743	Sdc3	0.649098334	0.011609386	-1.269804001	0.005337119	0.215542357
ENSMUSG00000041481	Serpina3g	2.190829457	1.515371799	-0.625829925	0.005716327	0.219815738
ENSMUSG00000072620	Slfn2	2.978663766	2.177903301	-0.691916456	0.006769416	0.233433153
ENSMUSG00000054404	Slfn5	2.464472562	0.406666293	-1.223302631	0.00695244	0.233433153
ENSMUSG00000035208	Slfn8	2.455872719	1.319837156	-0.588642527	0.00741022	0.240253008
ENSMUSG00000026104	Stat1	3.663134664	2.902057526	1.001482955	0.008575855	0.24802043
ENSMUSG00000078921	Tgtp2	0.980352131	0.354522206	-0.589900777	0.00893012	0.248828807
ENSMUSG00000044583	Tlr7	1.462124564	0.508475146	-0.630670559	0.00910955	0.24889219
ENSMUSG00000096780	Tmem181b-ps	0.121501003	0.721974762	-0.580269592	0.01220133	0.274798325
ENSMUSG00000030921	Trim30a	3.066419399	2.18736237	-0.835507176	0.013251235	0.286599569
ENSMUSG00000057596	Trim30d	1.683545313	1.014707115	-0.603147419	0.021548705	0.349324397
ENSMUSG00000032175	Tyk2	2.623989179	1.179992767	-0.675457658	0.028949762	0.3881613
ENSMUSG00000102049	Zbed6	0.675768237	0.095498645	0.621099836	0.031003789	0.399317843
ENSMUSG00000027514	Zbp1	1.387453979	0.705883847	-1.651314986	0.034291585	0.415980392

Table S4. Differentially expressed genes (DEGs) of the marginal zone (MZ) cells between WT and *Tyk2*$^{-/-}$ mice and their respective fold-changes (FC), *P*-values and false discovery rate (FDR).

Transcipt ID	Gene name	log_2(WT_MZ)	log_2(KO_MZ)	log_2(FC)	*P*-value	FDR < 0.05
ENSMUSG00000033355	Rtp4	1.630481261	0.057975402	-1.572505859	8.68365E-11	1.68923E-06
ENSMUSG00000111083	AC153954.2	0.105805044	0.80362482	0.697819776	1.29438E-07	0.000629487
ENSMUSG00000037849	Ifi206	2.406872534	1.274112202	-1.132760332	1.1903E-07	0.000629487
ENSMUSG00000062488	Ifit3b	0.963477983	0.024513624	-0.938964359	3.95103E-07	0.001537186
ENSMUSG00000046841	Ckap4	0.537823666	1.762607004	1.224783338	1.7783E-06	0.003139609
ENSMUSG00000025888	Casp1	2.555576381	1.864033645	-0.691542736	4.2921E-06	0.003258673
ENSMUSG00000029561	Oasl2	0.663315716	0.046089353	-0.617226363	7.25168E-06	0.003258673
ENSMUSG00000057143	Trim12c	2.166684475	1.35807467	-0.808609805	3.086E-06	0.003258673
ENSMUSG00000030107	Usp18	1.241045757	0.037130757	-1.203915	3.6586E-06	0.003258673
ENSMUSG00000064215	Ifi27	2.405677672	0.640173452	-1.76550422	7.85543E-06	0.003321993
ENSMUSG00000033880	Lgals3bp	1.746671821	0.23545555	-1.511216271	9.75737E-06	0.003668558
ENSMUSG00000032175	Tyk2	2.440512309	1.434091712	-1.006420597	1.04391E-05	0.003692217
ENSMUSG00000114203	AC154849.1	0.62694192	1.537416615	0.910474695	1.06972E-05	0.003715925
ENSMUSG00000044583	Tlr7	1.90432215	1.052807904	-0.851514246	1.35558E-05	0.00405693
ENSMUSG00000079017	Ifi27l2a	1.675448364	0.07133685	-1.604111515	1.55608E-05	0.004287355
ENSMUSG00000074896	Ifit3	2.102777502	0.214094816	-1.888682686	1.63044E-05	0.004287355
ENSMUSG00000040511	Pvr	1.713577924	2.45299069	0.739412767	1.65297E-05	0.004287355
ENSMUSG00000017707	Serinc3	3.500345272	4.594567339	1.094222067	2.07139E-05	0.005165991
ENSMUSG00000026104	Stat1	4.564655015	3.575045401	-0.989609614	2.45104E-05	0.005744581
ENSMUSG00000073491	Ifi213	2.637114546	1.158739447	-1.478375099	2.51118E-05	0.005815483
ENSMUSG00000070327	Rnf213	2.303868482	1.457381954	-0.846486528	2.85123E-05	0.006232011
ENSMUSG00000000386	Mx1	0.661806002	0.006184005	-0.655621997	2.91612E-05	0.006303025
ENSMUSG00000105263	Gm42427	3.478218919	5.588073269	2.10985435	3.26227E-05	0.006823767
ENSMUSG00000066677	Ifi208	2.071442107	1.319755407	-0.751686699	3.72036E-05	0.006985043
ENSMUSG00000050668	Gpatch11	2.076375895	1.352197875	-0.724178021	3.89325E-05	0.007078074
ENSMUSG00000105353	Gm42428	2.575347862	3.766253957	1.190906094	3.95506E-05	0.007123867
ENSMUSG00000027514	Zbp1	1.744228682	0.851499949	-0.892728733	4.11598E-05	0.007196458
ENSMUSG00000047412	Zbtb44	1.381707411	2.076474592	0.694767181	4.43526E-05	0.007464264
ENSMUSG00000010358	Ifi35	2.973170609	2.140757228	-0.832413381	5.09553E-05	0.007804985
ENSMUSG00000032690	Oas2	1.318771084	0.012373713	-1.306397371	5.16811E-05	0.007854318
ENSMUSG00000104713	Gbp6	1.447705169	0.051515608	-1.396189561	5.32402E-05	0.008028538
ENSMUSG00000026222	Sp100	4.762046649	4.123647894	-0.638398754	7.06771E-05	0.009112424
ENSMUSG00000041827	Oasl1	1.217259477	0.206392731	-1.010866747	7.69449E-05	0.009719541
ENSMUSG00000002325	Irf9	2.964104729	1.865459097	-1.098645632	0.000100786	0.011201149
ENSMUSG00000110537	Gm4316	2.720277562	4.127152437	1.406874874	0.000105083	0.011231797
ENSMUSG00000072772	Grcc10	2.76944734	2.14173279	-0.62771455	0.000104454	0.011231797
ENSMUSG00000035208	Slfn8	1.67331396	0.682023454	-0.991290505	0.000118497	0.011939995

Transcipt ID	Gene name	$\log_2$(WT_MZ)	$\log_2$(KO_MZ)	$\log_2$(FC)	*P*-value	FDR < 0.05
ENSMUSG00000006360	Crip1	3.100077422	2.500300598	-0.599776824	0.000125983	0.0122537
ENSMUSG00000037921	Ddx60	2.293439636	0.466953432	-1.826486204	0.000139103	0.012858981
ENSMUSG00000110386	Gm42031	4.817351733	5.885325009	1.067973276	0.00014914	0.013068559
ENSMUSG00000033538	Casp4	1.130580445	0.511101359	-0.619479086	0.000151363	0.013100362
ENSMUSG00000024675	Ms4a4c	2.446030489	1.708359668	-0.737670822	0.0001546	0.013100362
ENSMUSG00000027397	Slc20a1	3.771017466	4.535625733	0.764608268	0.000155869	0.013100362
ENSMUSG00000046805	Mpeg1	1.717482098	1.124279756	-0.593202342	0.000161297	0.013307867
ENSMUSG00000078921	Tgtp2	2.365675386	0.883838522	-1.481836863	0.000169356	0.013653818
ENSMUSG00000027189	Trim44	1.60578658	2.234119368	0.628332788	0.000180091	0.013919107
ENSMUSG00000105703	Gm43305	6.763365858	7.632453293	0.869087435	0.000197509	0.014553599
ENSMUSG00000078193	Gm2000	5.009914961	3.958300928	-1.051614032	0.000215219	0.015448946
ENSMUSG00000062997	Rpl35	5.24624856	4.249631479	-0.996617081	0.000220319	0.015566224
ENSMUSG00000037443	Cep85	4.796574731	5.411362296	0.614787564	0.000231585	0.016032137
ENSMUSG00000069049	Eif2s3y	0.426810484	1.918403081	1.491592597	0.000262687	0.016888597
ENSMUSG00000037321	Tap1	3.731616699	3.089669284	-0.641947414	0.000277466	0.017524501
ENSMUSG00000044703	Phf11a	0.889014831	0.195082392	-0.693932439	0.000299917	0.018062818
ENSMUSG00000079435	Rpl36a	3.306960095	2.491072106	-0.815887988	0.000314639	0.018741806
ENSMUSG00000040483	Xaf1	1.574419697	0.46046826	-1.113951438	0.000320571	0.018954626
ENSMUSG00000105987	AI506816	3.146411453	3.758610816	0.612199363	0.000340673	0.019798166
ENSMUSG00000052776	Oas1a	1.007547929	0	-1.007547929	0.000340944	0.019798166
ENSMUSG00000030921	Trim30a	2.490007122	1.46680258	-1.023204542	0.000348277	0.019951031
ENSMUSG00000047735	Samd9l	2.942383598	2.246468109	-0.69591549	0.000364791	0.0202173
ENSMUSG00000090137	Uba52	3.929959173	2.970581249	-0.959377924	0.000394665	0.021034012
ENSMUSG00000020279	Il9r	4.117796009	3.080695911	-1.037100099	0.000397338	0.021118626
ENSMUSG00000075602	Ly6a	4.376523317	2.729092067	-1.64743125	0.000413771	0.021325413
ENSMUSG00000096768	Erdr1	2.317501598	4.304865433	1.987363834	0.000423738	0.02150966
ENSMUSG00000044712	Slc38a6	2.297578077	2.90115966	0.603581584	0.000440101	0.021840036
ENSMUSG00000094796	BC147527	1.821279939	0.952568941	-0.868710998	0.000513405	0.023597982
ENSMUSG00000000275	Trim25	2.167000238	1.562593373	-0.604406865	0.000510717	0.023597982
ENSMUSG00000045932	Ifit2	1.014744725	0.287700662	-0.727044063	0.000579373	0.025266185
ENSMUSG00000096979	Gm26880	2.851980552	3.931170606	1.079190054	0.000590802	0.025370583
ENSMUSG00000046879	Irgm1	2.649265858	1.767611633	-0.881654226	0.000598467	0.025474788
ENSMUSG00000022906	Parp9	1.745053738	1.031426639	-0.713627099	0.000604295	0.025499677
ENSMUSG00000096255	Dynlt1b	0.994242321	1.794841036	0.800598715	0.000651127	0.026610022
ENSMUSG00000060802	B2m	6.571564669	5.610052449	-0.96151222	0.000702118	0.027592528
ENSMUSG00000007892	Rplp1	4.221519356	3.460837768	-0.760681589	0.00072806	0.027784653
ENSMUSG00000072621	Slfn10-ps	0.750707172	1.391466091	0.640758919	0.000730391	0.027784653
ENSMUSG00000096780	Tmem181b-ps	0.319148762	1.116496124	0.797347362	0.000732716	0.027784653
ENSMUSG00000038274	Fau	3.866383664	3.01052532	-0.855858345	0.000780517	0.029031362
ENSMUSG00000078920	Ifi47	2.039926655	1.345697442	-0.694229213	0.000826509	0.030172337
ENSMUSG00000022708	Zbtb20	3.182858401	3.801568016	0.618709615	0.000842714	0.03041431
ENSMUSG00000024079	Eif2ak2	1.8249556	1.135568912	-0.689386688	0.000849451	0.030600668

Transcipt ID	Gene name	log_2(WT_MZ)	log_2(KO_MZ)	log_2(FC)	*P*-value	FDR < 0.05
ENSMUSG00000016252	Atp5e	2.308564646	1.620100469	-0.688464177	0.000857227	0.030823726
ENSMUSG00000091649	Phf11b	1.840043898	1.157276129	-0.682767769	0.000874415	0.030983605
ENSMUSG00000063457	Rps15	3.615331113	2.773486426	-0.841844687	0.000950055	0.031974778
ENSMUSG00000029823	Luc7l2	3.825269502	4.616190343	0.790920841	0.000964521	0.032238521
ENSMUSG00000062783	Csprs	2.362800273	1.543430287	-0.819369986	0.000978769	0.032380956
ENSMUSG00000046718	Bst2	1.218878526	0.616199561	-0.602678965	0.000991394	0.032508304
ENSMUSG00000056501	Cebpb	2.335996326	3.405486195	1.069489868	0.001030911	0.032822114
ENSMUSG00000082286	Pisd-ps1	1.662002721	2.679444564	1.017441842	0.001087236	0.034057989
ENSMUSG00000020638	Cmpk2	1.377087302	0.433432103	-0.943655199	0.001114687	0.034473781
ENSMUSG00000037563	Rps16	4.293891866	3.414408004	-0.879483862	0.001113046	0.034473781
ENSMUSG00000043263	Ifi209	3.922225006	3.323884994	-0.598340012	0.00117209	0.034757115
ENSMUSG00000038642	Ctss	5.388315092	4.802796498	-0.585518594	0.001177193	0.034782671
ENSMUSG00000062006	Rpl34	4.214358439	3.529868524	-0.684489914	0.001194284	0.034883486
ENSMUSG00000041841	Rpl37	2.419201987	1.592329174	-0.826872813	0.001213884	0.035037258
ENSMUSG00000030256	Bhlhe41	1.870052621	2.808476223	0.938423602	0.001220987	0.035135883
ENSMUSG00000001123	Lgals9	2.999547803	2.19723476	-0.802313043	0.001244132	0.035591326
ENSMUSG00000022014	Epsti1	2.017626602	1.336683252	-0.68094335	0.001369268	0.037128085
ENSMUSG00000041481	Serpina3g	3.105483969	2.218863936	-0.886620033	0.001395884	0.037380879
ENSMUSG00000057322	Rpl38	2.915574033	2.095912512	-0.819661521	0.001414343	0.037486998
ENSMUSG00000079523	Tmsb10	5.600974118	4.925433975	-0.675540143	0.001417594	0.037486998
ENSMUSG00000046330	Rpl37a	4.013385288	2.976072734	-1.037312554	0.001464691	0.037939598
ENSMUSG00000012848	Rps5	4.765835593	4.027429766	-0.738405827	0.001490677	0.038132922
ENSMUSG00000012405	Rpl15	5.123454132	4.362699126	-0.760755006	0.001508445	0.038170012
ENSMUSG00000098274	Rpl24	3.487272984	2.603238575	-0.88403441	0.001513397	0.0382339
ENSMUSG00000106734	Gm20559	2.420113893	1.811563158	-0.608550735	0.001533397	0.038439646
ENSMUSG00000058546	Rpl23a	4.463785475	3.68142799	-0.782357485	0.001610434	0.039031936
ENSMUSG00000003429	Rps11	4.863355235	4.115185306	-0.748169929	0.001617522	0.039031936
ENSMUSG00000063882	Uqcrh	2.708989636	1.994082081	-0.714907555	0.001612683	0.039031936
ENSMUSG00000021200	Asb2	3.099724283	2.318737985	-0.780986297	0.00166871	0.039663802
ENSMUSG00000017830	Dhx58	1.221836272	0.605769721	-0.616066551	0.00170564	0.040021191
ENSMUSG00000027091	Zc3h15	2.44601907	3.04575502	0.599735949	0.001743704	0.040574479
ENSMUSG00000057841	Rpl32	6.35821918	5.296410296	-1.061808884	0.001751286	0.040655803
ENSMUSG00000079641	Rpl39	2.390022911	1.437423737	-0.952599173	0.001781127	0.040955386
ENSMUSG00000061477	Rps7	4.926760926	4.0410226	-0.885738326	0.001829586	0.041529675
ENSMUSG00000022216	Psme1	3.040238995	2.261299618	-0.778939377	0.001871084	0.041837005
ENSMUSG00000025794	Rpl14	4.708321593	3.967339113	-0.74098248	0.00187675	0.04188829
ENSMUSG00000019984	Med23	1.620658586	2.239758508	0.619099922	0.00203134	0.043567428
ENSMUSG00000024677	Ms4a6b	2.337917787	1.744552466	-0.593365321	0.002037109	0.043587571
ENSMUSG00000000682	Cd52	4.932468392	4.07012698	-0.862341411	0.002053577	0.043659263
ENSMUSG00000025743	Sdc3	0.608691522	0.019095496	-0.589596025	0.002108801	0.044015564
ENSMUSG00000043716	Rpl7	4.32613367	3.640346496	-0.685787174	0.002141733	0.044299491
ENSMUSG00000090523	Gypc	2.15069257	1.563144231	-0.587548339	0.002169958	0.044495295

Transcipt ID	Gene name	log_2(WT_MZ)	log_2(KO_MZ)	log_2(FC)	*P*-value	FDR < 0.05
ENSMUSG00000045128	Rpl18a	6.355997155	5.307471137	-1.048526019	0.00217067	0.044495295
ENSMUSG00000087528	9830144P21Rik	1.735078315	2.456798889	0.721720574	0.002187604	0.044795216
ENSMUSG00000090733	Rps27	6.125036132	5.238079743	-0.88695639	0.002203754	0.045010913
ENSMUSG00000029322	Plac8	4.727725415	3.910756856	-0.816968559	0.002294366	0.045755086
ENSMUSG00000040952	Rps19	6.084573489	5.132526494	-0.952046995	0.002350121	0.046168081
ENSMUSG00000074129	Rpl13a	5.533212797	4.719832668	-0.81338013	0.002363879	0.046308703
ENSMUSG00000041453	Rpl21	4.638895239	3.961927368	-0.676967871	0.00240456	0.046312784
ENSMUSG00000060036	Rpl3	5.499710228	4.876644925	-0.623065303	0.002370803	0.046312784
ENSMUSG00000070501	Ifi214	1.894013647	1.245990199	-0.648023448	0.002471101	0.04723441
ENSMUSG00000063316	Rpl27	2.665616619	2.013681585	-0.651935034	0.002505312	0.047408397
ENSMUSG00000025362	Rps26	3.445709172	2.552606304	-0.893102869	0.00255081	0.047942898
ENSMUSG00000078606	Gm4070	2.048399956	0.316073438	-1.732326518	0.002553822	0.04795318
ENSMUSG00000037754	Ppp1r16b	5.091278847	5.744743318	0.653464471	0.002726106	0.049794313
ENSMUSG00000039997	Ifi203	3.446894961	2.828433844	-0.618461117	0.002750731	0.05002865
ENSMUSG00000046364	Rpl27a	3.41358776	2.713813228	-0.699774531	0.002843457	0.050746574
ENSMUSG00000024423	Impact	4.970621703	5.648112412	0.677490709	0.002850169	0.050752578
ENSMUSG00000017404	Rpl19	4.541935309	3.785777332	-0.756157978	0.003047556	0.052844842
ENSMUSG00000071644	Eef1g	4.707566956	4.050374684	-0.657192272	0.003090987	0.053149147
ENSMUSG00000053565	Eif3k	3.479880404	2.875756484	-0.60412392	0.003131072	0.053616852
ENSMUSG00000073702	Rpl31	3.349536452	2.74168115	-0.607855302	0.003156851	0.053843635
ENSMUSG00000020460	Rps27a	4.513612625	3.693534364	-0.820078262	0.003336917	0.055245145
ENSMUSG00000046962	Zbtb21	2.536893794	3.282723113	0.74582932	0.003345306	0.055336944
ENSMUSG00000059291	Rpl11	3.944783125	3.108002976	-0.836780148	0.003465201	0.056220653
ENSMUSG00000090862	Rps13	3.631651832	2.894965975	-0.736685856	0.003688505	0.058189006
ENSMUSG00000030744	Rps3	5.005382512	4.32683488	-0.678547632	0.003690289	0.058189006
ENSMUSG00000008683	Rps15a	3.737826742	2.850797923	-0.887028819	0.003735061	0.058453857
ENSMUSG00000047215	Rpl9	4.976139315	4.147879694	-0.828259622	0.003818185	0.059153744
ENSMUSG00000068457	Uty	0.550618299	1.5057079	0.955089601	0.003810577	0.059153744
ENSMUSG00000028495	Rps6	5.663888644	4.867000991	-0.796887653	0.003854933	0.059303847
ENSMUSG00000034892	Rps29	3.45067027	2.669976043	-0.780694227	0.003864304	0.05935009
ENSMUSG00000026034	Clk1	5.541539918	6.192220396	0.650680477	0.003963827	0.060053213
ENSMUSG00000076501	Igkv2-137	2.160104343	1.449294877	-0.710809466	0.004056754	0.060647186
ENSMUSG00000049775	Tmsb4x	6.631510746	5.851062592	-0.780448154	0.004085514	0.060668322
ENSMUSG00000060636	Rpl35a	3.27475817	2.640477062	-0.634281108	0.004175055	0.061301299
ENSMUSG00000006333	Rps9	5.118134397	4.328427653	-0.789706745	0.004178337	0.061301299
ENSMUSG00000026643	Nmt2	3.16746947	3.770816322	0.603346852	0.004229544	0.061677152
ENSMUSG00000025498	Irf7	1.760500893	0.86705255	-0.893448343	0.00426378	0.061944215
ENSMUSG00000036751	Cox6b1	2.243101738	1.653457555	-0.589644183	0.00432624	0.062363937
ENSMUSG00000015165	Hnrnpl	3.990826288	4.584363427	0.593537139	0.004351838	0.062569333
ENSMUSG00000049517	Rps23	4.510376589	3.648458727	-0.861917862	0.004389109	0.062869149
ENSMUSG00000000740	Rpl13	6.48594878	5.566679091	-0.919269689	0.004410339	0.062945206
ENSMUSG00000008668	Rps18	6.022972976	5.183809752	-0.839163224	0.004461779	0.063384037

Transcipt ID	Gene name	$\log_2$(WT_MZ)	$\log_2$(KO_MZ)	$\log_2$(FC)	*P*-value	FDR < 0.05
ENSMUSG00000024608	Rps14	5.53418584	4.821872147	-0.712313693	0.004518971	0.063654993
ENSMUSG00000024353	Mzb1	4.634064255	4.052925698	-0.581138557	0.004683822	0.064620137
ENSMUSG00000093674	Rpl41	4.453387214	3.492457904	-0.96092931	0.004794983	0.065553967
ENSMUSG00000062328	Rpl17	3.571664731	2.89071145	-0.680953281	0.004862352	0.066191273
ENSMUSG00000003970	Rpl8	4.196997847	3.499119985	-0.697877863	0.005042538	0.067094733
ENSMUSG00000031818	Cox4i1	4.192819739	3.493813489	-0.699006249	0.005084968	0.067474677
ENSMUSG00000028234	Rps20	3.705669175	2.981676462	-0.723992713	0.005092199	0.06748257
ENSMUSG00000072692	Rpl37rt	2.97342273	2.176430534	-0.796992196	0.005114494	0.067602013
ENSMUSG00000067274	Rplp0	6.026251366	5.284681671	-0.741569695	0.005155099	0.067987898
ENSMUSG00000048758	Rpl29	3.264766161	2.614453968	-0.650312193	0.005230275	0.068534226
ENSMUSG00000029614	Rpl6	4.730913032	4.006351882	-0.72456115	0.005300209	0.068955421
ENSMUSG00000045826	Ptprcap	3.693035493	3.110992134	-0.582043359	0.005324389	0.069034724
ENSMUSG00000031723	Txnl4b	1.253847366	1.840138914	0.586291548	0.005403087	0.069791669
ENSMUSG00000025290	Rps24	3.969488709	3.310850185	-0.658638524	0.005707391	0.071056279
ENSMUSG00000032449	Slc25a36	3.678093018	4.269876008	0.59178299	0.005752071	0.071094904
ENSMUSG00000073640	Rpl27-ps3	3.154193561	2.085209834	-1.068983727	0.006240979	0.07416636
ENSMUSG00000072620	Slfn2	2.109685303	1.317336156	-0.792349148	0.006320882	0.074680664
ENSMUSG00000027487	Cdk5rap1	0.913876655	1.599406034	0.685529379	0.006422377	0.075171179
ENSMUSG00000061787	Rps17	2.609872597	1.954020078	-0.655852519	0.006460584	0.075482125
ENSMUSG00000068240	Gm11808	3.872524283	2.904110968	-0.968413314	0.006662824	0.076738846
ENSMUSG00000018476	Kdm6b	4.860984622	5.557684852	0.69670023	0.006933948	0.078465695
ENSMUSG00000056429	Tgoln1	5.023702104	5.75869268	0.734990576	0.007235494	0.080109317
ENSMUSG00000103696	Gm37531	4.331913877	5.310711286	0.978797409	0.00727049	0.080243375
ENSMUSG00000085148	Mir22hg	1.748428972	2.365043791	0.616614819	0.007576778	0.081878702
ENSMUSG00000057863	Rpl36	3.827247701	2.856012525	-0.971235176	0.007605444	0.081966038
ENSMUSG00000095041	AC149090.1	4.923096266	6.067784055	1.144687789	0.007709645	0.082358988
ENSMUSG00000025279	Dnase1l3	4.510643796	5.266678409	0.756034613	0.008005154	0.084206447
ENSMUSG00000028081	Rps3a1	4.772588429	4.185597166	-0.586991263	0.00801343	0.084206447
ENSMUSG00000067288	Rps28	3.21737431	2.477210984	-0.740163326	0.008688909	0.087036743
ENSMUSG00000059070	Rpl18	3.994487064	3.2966468	-0.697840263	0.009077231	0.08940728
ENSMUSG00000044533	Rps2	5.146549206	4.427187384	-0.719361822	0.009741575	0.092911306
ENSMUSG00000037805	Rpl10a	3.976311258	3.389625324	-0.586685934	0.01003786	0.094108072
ENSMUSG00000086583	Gm15500	5.363375252	4.751075703	-0.61229955	0.010172195	0.094588767
ENSMUSG00000032803	Cdv3	5.020930963	5.762796043	0.74186508	0.010627984	0.096361161
ENSMUSG00000052040	Klf13	4.707717731	5.331976234	0.624258503	0.010660749	0.096457461
ENSMUSG00000034634	Ly6d	6.54672361	5.89168931	-0.6550343	0.010942492	0.097778732
ENSMUSG00000061589	Dot1l	2.183489919	2.789471529	0.60598161	0.011458124	0.100222522
ENSMUSG00000106044	Gm42860	2.523908701	3.162656616	0.638747915	0.011676028	0.101218258
ENSMUSG00000032035	Ets1	6.384747525	5.777422506	-0.607325019	0.011710712	0.101293229
ENSMUSG00000040270	Bach2	1.717554717	2.527577687	0.810022969	0.011904792	0.102154351
ENSMUSG00000096954	Gdap10	5.16677996	6.357334037	1.190554077	0.012254603	0.103814978
ENSMUSG00000044734	Serpinb1a	3.074404347	2.427448234	-0.646956113	0.012551209	0.104924224

Transcipt ID	Gene name	log_2(WT_MZ)	log_2(KO_MZ)	log_2(FC)	*P*-value	FDR < 0.05
ENSMUSG00000061315	Naca	3.827559254	3.24721759	-0.580341665	0.012782619	0.105948144
ENSMUSG00000029178	Klf3	2.186364918	2.766527362	0.580162444	0.013228911	0.107809806
ENSMUSG00000034120	Srsf2	4.941011714	5.683904188	0.742892474	0.013286611	0.107927129
ENSMUSG00000024334	H2-Oa	3.852377381	3.214247846	-0.638129535	0.013354847	0.108204483
ENSMUSG00000052423	B4galt3	2.766432582	3.374467542	0.60803496	0.014381809	0.113083807
ENSMUSG00000022048	Dpysl2	2.957366775	3.749665781	0.792299006	0.014860936	0.115405106
ENSMUSG00000008682	Rpl10	4.323818851	3.671841738	-0.651977113	0.01758293	0.125289648
ENSMUSG00000113476	AC154517.3	2.906334067	3.486533313	0.580199246	0.017778237	0.125577359
ENSMUSG00000062647	Rpl7a	3.902663934	3.269895562	-0.632768372	0.017857154	0.125906205
ENSMUSG00000031258	Xkrx	1.007367113	1.747309783	0.739942669	0.017941405	0.126180095
ENSMUSG00000021701	Plk2	4.217400945	4.908674403	0.691273459	0.01812177	0.12694375
ENSMUSG00000033685	Ucp2	3.965011263	3.329496427	-0.635514836	0.018250993	0.127234563
ENSMUSG00000033364	Usp37	2.574214887	3.270282819	0.696067932	0.018880576	0.129819856
ENSMUSG00000024063	Lbh	3.382011305	3.977547217	0.595535911	0.019920311	0.133303652
ENSMUSG00000032602	Slc25a20	2.166761777	1.439081874	-0.727679903	0.020906658	0.136234371
ENSMUSG00000094006	Igkv4-59	2.135906769	1.441488159	-0.69441861	0.021191675	0.137459706
ENSMUSG00000112822	AC126943.1	1.343210156	2.265198121	0.921987965	0.022463921	0.141764359
ENSMUSG00000076940	Iglv2	3.581947183	2.822099186	-0.759847997	0.022853893	0.142705718
ENSMUSG00000021754	Map3k1	5.07903905	5.71373048	0.634691429	0.023631041	0.145102079
ENSMUSG00000103867	9630010A21Rik	1.684992889	2.403594309	0.71860142	0.026574669	0.153947897
ENSMUSG00000103996	4833421G17Rik	1.480484851	2.166516071	0.68603122	0.027146468	0.155424411
ENSMUSG00000094472	Gm21897	1.427687267	2.177165921	0.749478654	0.027511051	0.156574742
ENSMUSG00000089726	Mir17hg	1.125188867	1.744663827	0.61947496	0.02901729	0.16118599
ENSMUSG00000038375	Trp53inp2	4.008997378	4.990982141	0.981984763	0.029844354	0.163995892
ENSMUSG00000030213	Atf7ip	4.358315644	4.952128338	0.593812694	0.031266277	0.16726321
ENSMUSG00000036432	Siah2	1.886062396	2.489502999	0.603440603	0.032023425	0.169096549
ENSMUSG00000107041	Gm42735	1.528429464	2.18021442	0.651784956	0.03239381	0.170226111
ENSMUSG00000103591	Gm38365	2.873442697	3.819573569	0.946130871	0.033650273	0.173524175
ENSMUSG00000056579	Tug1	4.575290054	5.226153856	0.650863802	0.034161702	0.174376171
ENSMUSG00000015597	Zfp318	2.234059805	2.873836804	0.639776998	0.034133641	0.174376171
ENSMUSG00000020275	Rel	6.334359772	7.028371128	0.694011356	0.03439274	0.175105419
ENSMUSG00000107362	Gm40309	2.767310808	3.516488757	0.749177949	0.034939118	0.176629092
ENSMUSG00000098814	Igkv19-93	2.199621053	1.066415488	-1.133205566	0.035361148	0.177700961
ENSMUSG00000026915	Strbp	3.454316748	4.108123348	0.6538066	0.035344501	0.177700961
ENSMUSG00000025451	Paip1	1.571030263	2.297791962	0.726761699	0.03582969	0.178952994
ENSMUSG00000108803	4930533N22Rik	1.224582675	1.832567419	0.607984744	0.037123588	0.182136937
ENSMUSG00000050299	Gm9843	2.544776154	1.743795643	-0.800980511	0.038393083	0.185878705
ENSMUSG00000074698	Csnk2a1	4.784805565	5.38508739	0.600281825	0.039167361	0.187158604
ENSMUSG00000025283	Sat1	5.23612275	5.922655832	0.686533081	0.039294495	0.187408493
ENSMUSG00000104339	C130089K02Rik	0.838692594	1.52446205	0.685769455	0.041356558	0.193066744
ENSMUSG00000030059	Tmf1	3.647781161	4.25954591	0.611764749	0.043164423	0.19808387

Table S5. Differentially expressed genes characteristic of TYK2 deficiency shared in both FO and MZ cells and their respective fold-changes and *P*-values.

Transcipt ID	Gene name	Follicular		Marginal zone	
		$\log_2$(FC)	*P*-value	$\log_2$(FC)	*P*-value
ENSMUSG00000095041	AC149090.1	1.014076426	0.000377501	1.144687789	0.007709645
ENSMUSG00000105987	AI506816	0.590192805	0.000988124	0.612199363	0.000340673
ENSMUSG00000025888	Casp1	-0.614889658	0.001644965	-0.691542736	4.2921E-06
ENSMUSG00000033538	Casp4	-0.788584752	1.32307E-07	-0.619479086	0.000151363
ENSMUSG00000046841	Ckap4	0.78062608	0.000317226	1.224783338	1.7783E-06
ENSMUSG00000062783	Csprs	-0.721359418	0.0046022	-0.819369986	0.000978769
ENSMUSG00000037921	Ddx60	-0.705216956	1.0321E-05	-1.826486204	0.000139103
ENSMUSG00000017830	Dhx58	-0.730459288	4.32709E-06	-0.616066551	0.00170564
ENSMUSG00000096255	Dynlt1b	0.911982487	7.14071E-06	0.800598715	0.000651127
ENSMUSG00000096768	Erdr1	1.597643372	0.003022915	1.987363834	0.000423738
ENSMUSG00000104713	Gbp6	-0.736978175	0.001484622	-1.396189561	5.32402E-05
ENSMUSG00000078606	Gm4070	-1.723504347	0.000127761	-1.732326518	0.002553822
ENSMUSG00000110386	Gm42031	1.468757507	0.000218941	1.067973276	0.00014914
ENSMUSG00000105263	Gm42427	1.56667504	0.000103684	2.10985435	3.26227E-05
ENSMUSG00000110537	Gm4316	1.052243734	1.86191E-05	1.406874874	0.000105083
ENSMUSG00000105703	Gm43305	1.170540125	0.00014852	0.869087435	0.000197509
ENSMUSG00000037849	Ifi206	-0.951196866	0.000424255	-1.132760332	1.1903E-07
ENSMUSG00000066677	Ifi208	-0.806137011	0.001801663	-0.751686699	3.72036E-05
ENSMUSG00000073491	Ifi213	-1.887568421	3.79065E-06	-1.478375099	2.51118E-05
ENSMUSG00000070501	Ifi214	-0.589900777	0.00893012	-0.648023448	0.002471101
ENSMUSG00000064215	Ifi27	-1.137940342	4.02264E-06	-1.76550422	7.85543E-06
ENSMUSG00000079017	Ifi27l2a	-1.497775418	1.5288E-05	-1.604111515	1.55608E-05
ENSMUSG00000045932	Ifit2	-0.597377417	0.000842863	-0.727044063	0.000579373
ENSMUSG00000074896	Ifit3	-0.638394793	0.000264124	-1.888682686	1.63044E-05
ENSMUSG00000020279	Il9r	-1.404483721	3.10286E-08	-1.037100099	0.000397338
ENSMUSG00000025498	Irf7	-0.983013632	4.9925E-07	-0.893448343	0.00426378
ENSMUSG00000002325	Irf9	-0.715977595	0.000444487	-1.098645632	0.000100786
ENSMUSG00000046879	Irgm1	-0.598111585	0.00233851	-0.881654226	0.000598467
ENSMUSG00000033880	Lgals3bp	-0.720067956	5.68361E-05	-1.511216271	9.75737E-06
ENSMUSG00000075602	Ly6a	-1.879319384	0.000100242	-1.64743125	0.000413771
ENSMUSG00000000386	Mx1	-0.761909215	0.000229679	-0.655621997	2.91612E-05
ENSMUSG00000052776	Oas1a	-0.693673019	0.000236333	-1.007547929	0.000340944
ENSMUSG00000032690	Oas2	-1.127807319	5.86276E-05	-1.306397371	5.16811E-05
ENSMUSG00000041827	Oasl1	-1.116828203	4.55977E-08	-1.010866747	7.69449E-05
ENSMUSG00000029561	Oasl2	-0.730055686	6.8833E-05	-0.617226363	7.25168E-06
ENSMUSG00000082286	Pisd-ps1	0.729441634	0.000499973	1.017441842	0.001087236
ENSMUSG00000029322	Plac8	-0.734301719	6.84626E-05	-0.816968559	0.002294366
ENSMUSG00000070327	Rnf213	-1.174044461	7.68596E-06	-0.846486528	2.85123E-05

Transcipt ID	Gene name	Follicular		Marginal zone	
		**log_2(FC)**	***P*-value**	**log_2(FC)**	***P*-value**
ENSMUSG00000025743	Sdc3	-0.637488948	2.09748E-05	-0.589596025	0.002108801
ENSMUSG00000041481	Serpina3g	-0.675457658	0.028949762	-0.886620033	0.001395884
ENSMUSG00000072620	Slfn2	-0.800760465	0.00152012	-0.792349148	0.006320882
ENSMUSG00000035208	Slfn8	-1.136035563	4.84913E-05	-0.991290505	0.000118497
ENSMUSG00000026104	Stat1	-0.761077138	0.004970557	-0.989609614	2.45104E-05
ENSMUSG00000078921	Tgtp2	-0.625829925	0.005716327	-1.481836863	0.000169356
ENSMUSG00000044583	Tlr7	-0.953649418	1.47899E-05	-0.851514246	1.35558E-05
ENSMUSG00000096780	Tmem181b-ps	0.600473759	0.000108961	0.797347362	0.000732716
ENSMUSG00000030921	Trim30a	-0.87905703	0.000711356	-1.023204542	0.000348277
ENSMUSG00000032175	Tyk2	-1.443996413	1.05354E-07	-1.006420597	1.04391E-05

Table S6. Significance levels for proliferation and differentiation in the *in vitro* B cell cultures. IMIQ group was compared to the IFNα + IMIQ and CL097 group with IFNα + CL097, in WT and *Tyk2*$^{-/-}$, respectively with a two-tailed Student t-test (data shown in Fig. 5). Each comparison shows their respective significance level and *P*-value in brackets. ns = not statistically significant; * *P* < 0.05; ** *P* < 0.01; *** *P* < 0.001.

	**WT**		
	IMIQ *vs* IFNα + IMIQ	CL097 *vs* IFNα + CL097	C *vs* IFNα
**Proliferation**	* (0.037)	** (0.0021)	*** (0.0001)
**Differentiation (% CD138^{+})**	** (0.0069)	*** (0.00069)	* (0.02)

	***Tyk2*$^{-/-}$**		
	IMIQ *vs* IFNα + IMIQ	CL097 *vs* IFNα + CL097	C *vs* IFNα
**Proliferation**	*** (0.0001)	*** (0.0003)	* (0.02)
**Differentiation (% CD138^{+})**	*** (0.0010)	*** (0.0001)	ns (0.20)

Table S7. Significance levels achieved by *Tlr7* gene expression at 6 h. The control group was compared to the different treatments in WT and *Tyk2*$^{-/-}$ with a two-tailed Student t-test (data shown in Fig. 6). Each comparison shows their respective significance level and *P*-value in brackets. ns = not statistically significant; * *P* < 0.05; ** *P* < 0.01; *** *P* < 0.001

	C *vs* IMIQ	C *vs* CL097	C *vs* IFNα	C *vs* IFNα + IMIQ	C *vs* IFNα + CL097	C *vs* LPS	C *vs* antiCD40 + IL4
**WT**	ns (0.0519)	*** (0.0005)	*** (< 0.0001)	*** (0.0003)	*** (0.0004)	** (0.0018)	* (0.025)
***Tyk2*$^{-/-}$**	* (0.0211)	*** (0.0002)	** (0.001)	*** (0.0001)	*** (< 0.0001)	ns (0.5775)	*** (0.0001)

Table S8. Significance levels for the production of IL6 and IgG in the *in vitro* B cell cultures. IMIQ group was compared to IFNα + IMIQ and CL097 group with IFNα + CL097, in WT and *Tyk2*$^{-/-}$, respectively with a two-tailed Student t-test (data shown in Fig. 6). Each comparison shows their respective significance level and *P*-value in brackets. ns = not statistically significant; * *P* < 0.05; ** *P* < 0.01; *** *P* < 0.001.

	**WT**		
	IMIQ *vs* IFNα + IMIQ	CL097 *vs* IFNα + CL097	C *vs* IFNα
**IL6**	ns (0.99)	* (0.049)	ns (0.41)
**IgG**	** (0.025)	** (0.022)	ns (0.72)

	Tyk2$^{-/-}$		
	IMIQ *vs* IFNα + IMIQ	CL097 *vs* IFNα + CL097	C *vs* IFNα
**IL6**	ns (0.48)	ns (0.24)	ns (0.45)
**IgG**	** (0.0058)	** (0.0014)	ns (0.38)
